# Target-Based Antiviral Drug Development Against Human Respiratory Viruses

**DOI:** 10.3390/pathogens15090903

**Published:** 2026-08-27

**Authors:** Subodh Kumar Samrat, Gauri Srivastava, Ran Zhang, Zhong Li, Hongmin Li

**Affiliations:** 1 Department of Pharmacology and Toxicology, R. Ken Coit College of Pharmacy, The University of Arizona, 1703 E Mabel St, Tucson, AZ 85721, USA; gaurisri100@arizona.edu (G.S.);; 2 Department of Chemistry & Biochemistry, College of Science & College of Medicine, The University of Arizona, Tucson, AZ 85721, USA; 3 The BIO5 Institute, The University of Arizona, Tucson, AZ 85721, USA; 4 Department of Molecular & Cellular Biology, College of Science, The University of Arizona, Tucson, AZ 85721, USA

**Keywords:** respiratory viruses, antiviral development, drug discovery, viral replication, coronavirus, respiratory syncytial virus (RSV), adenovirus, human parainfluenza virus (hPIV), measles, antiviral review

## Abstract

Human respiratory viruses represent a major global health burden, causing millions of severe infections and deaths annually. Despite the central role of vaccines in prevention, their limitations, such as incomplete coverage, waning immunity, and vulnerability to viral evolution, underscore the urgent need for effective antiviral therapeutics. This review examines the principles and applications of target-based antiviral drug development against human respiratory viruses, emphasizing the identification and exploitation of conserved viral and host targets. Key viral proteins, including RNA-dependent RNA polymerases, proteases, and fusion glycoproteins, are analyzed across major virus families such as coronaviruses, paramyxoviruses, and adenoviruses, highlighting their structural features, functional constraints, and therapeutic potential. We further explore the integration of high-throughput screening and rational drug design, supported by advances in structural biology, cryo-electron microscopy, and computational approaches, including artificial intelligence-driven drug discovery. These methodologies collectively enhance the precision and efficiency of antiviral development. However, significant challenges remain, particularly the conflict between viral mutation and target conservation, the rapid emergence of drug resistance, and the safety limitations of host-targeted therapies. Finally, we discuss emerging strategies to overcome these barriers, including combination therapies and the development of broad-spectrum antivirals targeting conserved molecular mechanisms. This paper highlights a framework for the rational design of durable antiviral interventions capable of addressing both existing and emerging respiratory viral threats.

## 1. Introduction

Human respiratory viruses are among the most significant infectious disease threats due to their high transmissibility and capacity to cause severe lower respiratory tract infections (LRTIs). Acute lower respiratory tract infections (ALRTIs), many of which are viral, account for approximately 2.5 million deaths annually worldwide and are one of the most common causes of deaths occurring in children under the age of five [1]. Among these pathogens, respiratory syncytial virus (RSV), influenza virus, and severe acute respiratory syndrome coronavirus 2 (SARS-CoV-2) represent the greatest contributors to global morbidity and mortality.

This review focuses on the major human respiratory viruses for which antiviral drug development has achieved clinical success or demonstrates substantial therapeutic potential, including highly pathogenic and seasonal coronaviruses, influenza viruses, respiratory syncytial virus, rhinoviruses, human metapneumovirus, parainfluenza viruses, adenoviruses, and respiratory pathogens such as measles and rubella viruses. Together, these viruses account for a substantial proportion of global respiratory disease burden, encompass diverse viral families and replication strategies, and illustrate the range of viral targets currently being exploited for antiviral development. By comparing conserved molecular targets across these clinically important pathogens, this review highlights common principles underlying target-based antiviral discovery as well as virus-specific therapeutic challenges.

RSV is a leading cause of pediatric respiratory disease, responsible for an estimated 3.6 million hospitalizations and 100,000 deaths annually in children under five. In addition to pediatric populations, RSV poses a significant risk to the elderly, where its severity and hospitalization rates rival those of influenza [2].

Influenza virus remains a persistent global health challenge due to its ability to undergo antigenic drift and shift, resulting in seasonal epidemics and occasional pandemics. Annually, influenza causes 3–5 million cases of severe illness and between 290,000 and 650,000 deaths worldwide [3]. Historical pandemics, most notably the 1918 H1N1 outbreak, demonstrate the virus’s capacity for rapid global spread and extreme mortality [4]. The continual evolution of influenza viruses necessitates frequent vaccine reformulation and contributes to antiviral resistance, underscoring the need for therapeutics that target highly conserved viral proteins such as neuraminidase, the cap-dependent endonuclease within the polymerase complex, and the M2 ion channel.

While endemic human coronaviruses are major contributors to seasonal respiratory infections worldwide, the emergence of SARS-CoV-2 further underscored the pandemic potential of respiratory viruses. As of 2026, COVID-19 has resulted in over 770 million confirmed cases [5] and more than 7 million deaths globally [5], with excess mortality estimates suggesting a substantially higher true burden. Beyond direct health effects, the pandemic exposed critical vulnerabilities in healthcare systems and highlighted the consequences of delayed therapeutic and vaccine responses.

Rhinoviruses, although typically associated with self-limiting upper respiratory tract infections, are the most common cause of the common cold and account for a substantial proportion of respiratory illnesses worldwide [6]. Their extensive genetic diversity and the absence of licensed antiviral therapies have hindered both vaccine and drug development, while their role in exacerbating asthma and chronic obstructive pulmonary disease highlights their considerable clinical and economic impact [7].

Although vaccines are central to prevention, their effectiveness is limited by several factors. Vaccines remain unavailable or provide incomplete protection for several clinically important respiratory viruses, including seasonal coronaviruses, RSV, parainfluenza virus, human metapneumovirus, and many rhinovirus and adenovirus strains [8]. Even when available, vaccine-induced immunity may be incomplete or short-lived, particularly in elderly or immunocompromised populations [9]. Additionally, vaccine development timelines leave populations vulnerable during emerging outbreaks, as demonstrated during the early stages of the COVID-19 pandemic. The emergence of immune-evasive variants, such as SARS-CoV-2 Omicron sub-lineages, further reduces vaccine effectiveness [10].

Given these limitations, antiviral therapeutics are essential for treating active infections, reducing disease severity, and limiting transmission. However, current antiviral options for respiratory viruses remain limited and often suboptimal.

For RSV, there is no widely used antiviral for routine clinical treatment. Ribavirin, the only FDA-approved option, has restricted use due to toxicity, teratogenicity, and limited efficacy [11,12]. Despite decades of investigation, there are currently no approved antiviral therapies for rhinovirus infection. Numerous experimental compounds targeting the viral capsid, 3C protease, and host factors required for replication have demonstrated antiviral activity; however, clinical translation has been limited by extensive viral diversity, rapid emergence of resistance, and insufficient efficacy [13]. Influenza antivirals, including neuraminidase inhibitors such as oseltamivir and zanamivir and the cap-dependent endonuclease inhibitor baloxavir marboxil, are most effective when administered early in infection and show reduced benefit beyond 48 h of symptom onset [13,14]. Resistance has also emerged, with oseltamivir-resistant H1N1 strains reported globally [15], highlighting the continued need for next-generation inhibitors targeting conserved viral proteins.

The COVID-19 pandemic accelerated antiviral development, resulting in agents such as remdesivir, molnupiravir, and nirmatrelvir-ritonavir (Paxlovid). Despite their impact, each has limitations: remdesivir requires intravenous administration and demonstrates modest mortality benefit [16,17]; molnupiravir raises concerns regarding mutagenicity [18,19]; and Paxlovid presents significant drug–drug interactions and reports of viral rebound [20,21]. These challenges highlight the need for more effective, accessible, and broadly active antiviral therapies.

Antiviral development has historically followed two primary approaches: non-specific and target-based strategies. Non-specific approaches include broad-spectrum antivirals and host-directed therapies, such as interferons or agents targeting host replication pathways. While these strategies may provide activity across multiple viruses, they are often limited by toxicity, off-target effects, and unpredictable efficacy. Ribavirin, for example, interferes with viral RNA synthesis but is associated with hemolytic anemia and teratogenicity [22]. Host-targeted therapies may reduce resistance risk but frequently disrupt essential cellular processes, limiting their clinical applicability [23,24].

In contrast, target-based antiviral development has emerged as the dominant paradigm. This approach focuses on selectively inhibiting viral proteins essential for replication, such as proteases, polymerases, and entry glycoproteins. Target-based antivirals offer improved specificity, reduced toxicity, and greater potential for rational optimization [25]. Advances in structural biology, including X-ray crystallography and cryo-electron microscopy, have enabled detailed characterization of viral proteins, facilitating structure-guided drug design.

Successful examples of this strategy include remdesivir and nirmatrelvir, SARS-CoV-2 main protease (Mpro) inhibitors [26,27,28]; influenza neuraminidase inhibitors (oseltamivir) [5,29,30] and cap-dependent endonuclease inhibitors (baloxavir marboxil [31,32]); and fusion inhibitors and polymerase inhibitors currently under investigation for RSV and other respiratory viruses [33]. These agents demonstrate the effectiveness of targeting conserved viral machinery, although resistance and strain variability remain ongoing challenges.

## 2. Overview of Respiratory Virus Classification and Therapeutic Landscape

### 2.1. Respiratory Virus Families

**Figure 1:**
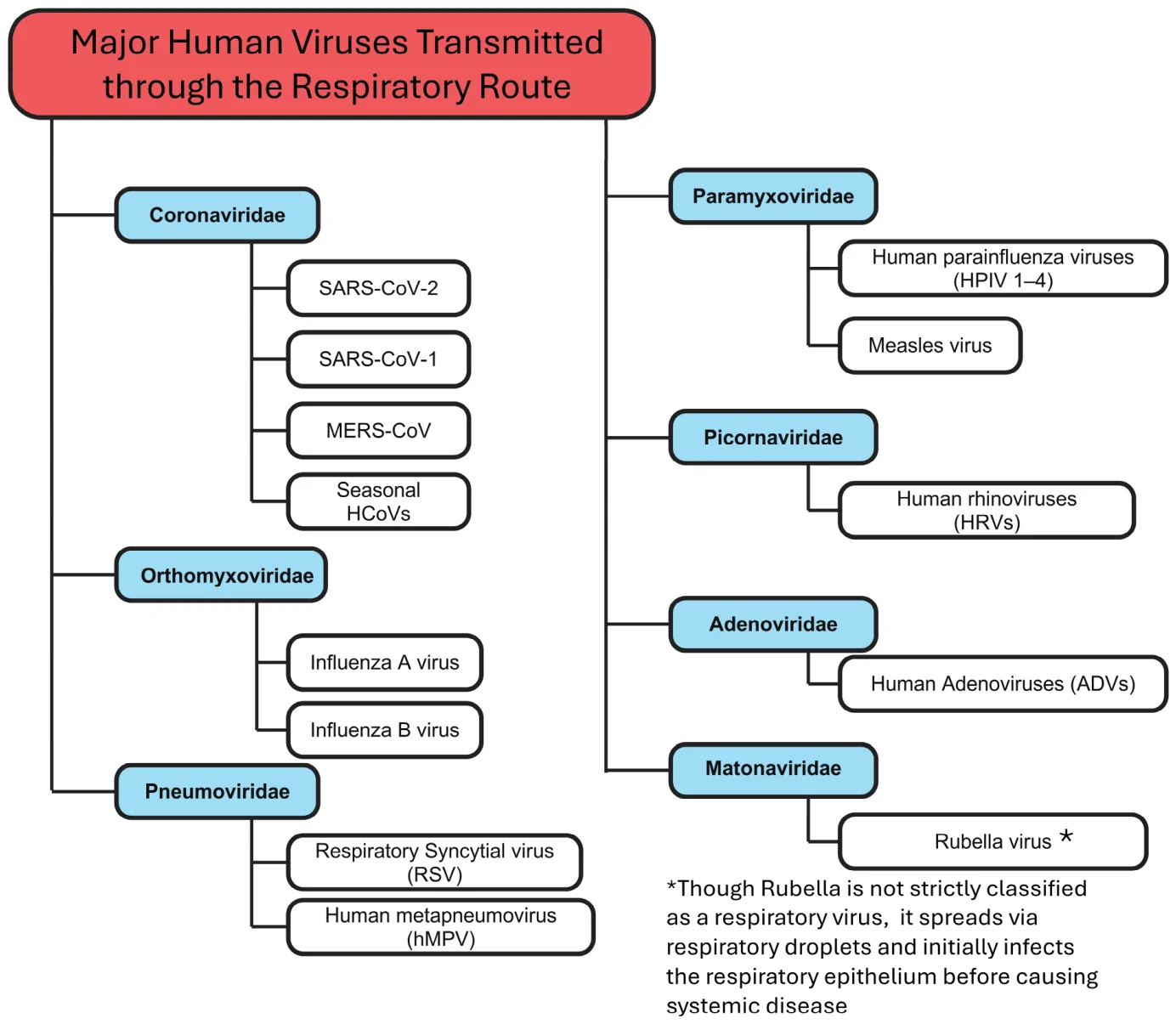
Taxonomic classification of the major human respiratory virus families discussed in this review. Respiratory viruses are classified into distinct viral families based on shared evolutionary relationships, genome organization, and replication strategies. The viruses included in this review comprise members of the *Coronaviridae*, *Orthomyxoviridae*, *Pneumoviridae*, *Paramyxoviridae*, *Picornaviridae*, *Adenoviridae*, and *Matonaviridae* families, encompassing the major respiratory pathogens and vaccine-preventable viruses considered in current antiviral development.

Human respiratory viruses comprise several genetically distinct viral families that differ in genome organization, replication strategy, and structural characteristics. Despite these differences, many exploit conserved molecular mechanisms that provide opportunities for antiviral intervention. Figure 1 outlines the major respiratory virus families discussed throughout this review.

### 2.2. Approved Antiviral Therapies and Promising Investigational Compounds for Human Respiratory Viruses

The current antiviral landscape for respiratory viruses remains highly heterogeneous. While several viruses, including SARS-CoV-2, influenza virus, and respiratory syncytial virus (RSV), have multiple approved or authorized therapeutics targeting distinct stages of the viral life cycle, most other respiratory viruses continue to lack virus-specific antiviral treatments. Consequently, current management of many respiratory infections remains primarily supportive despite continued advances in target-based antiviral discovery. Table 1 summarizes currently registered or approved antiviral agents and biologics for human respiratory viruses, whereas Table 2 highlights promising investigational compounds that target conserved viral or host factors discussed throughout this review.

## 3. Key Human Respiratory Viruses and Their Drug Targets

### 3.1. Coronaviruses

Coronaviruses, belonging to the family *Coronaviridae*, possess large (−30 kb), positive-sense RNA genomes encoding multiple structural and nonstructural proteins essential for replication and immune evasion (Figure 1) [34,35]. Recent structural reviews illuminate the architecture of key viral targets: spike (S), main protease (Mpro), papain-like protease (PLpro), and RNA-dependent RNA polymerase (RdRp). RdRp and Mpro are highly conserved, as their enzymatic functions are essential and their sequences remain largely unchanged across coronaviruses, making them attractive for broad-spectrum inhibitors (Table 3) [36,37,38]. In contrast, the spike (S) protein evolves rapidly under immune pressure and thus produces the fastest resistance (Table 3) [39,40]. High-value targets include RdRp and Mpro, whose well-defined, druggable pockets allow mechanism-based inhibition [36,41,42]. Surface-exposed S protein is readily accessible for antibodies or entry inhibitors, while intracellular enzymes like RdRp and the proteases require cell-permeable drugs [43,44,45]. In terms of structural dynamics, the S protein’s unstable prefusion conformation complicates vaccine and inhibitor design [46,47], while the proteases are relatively rigid, facilitating structure-guided drug discovery [48,49].

In addition to the highly pathogenic coronaviruses SARS-CoV, MERS-CoV, and SARS-CoV-2, four endemic human coronaviruses (HCoV-229E, HCoV-NL63, HCoV-OC43, and HCoV-HKU1) circulate globally and are responsible for a substantial proportion of seasonal upper respiratory tract infections [50]. These seasonal coronaviruses generally cause mild disease but can result in severe lower respiratory tract infections in older adults, infants, and immunocompromised individuals [50]. Although they differ in receptor usage, with HCoV-229E utilizing aminopeptidase N (APN), HCoV-NL63 binding ACE2, and HCoV-OC43 and HCoV-HKU1 recognizing 9-O-acetylated sialic acid receptors, their replication strategies remain highly conserved and rely on viral proteases (Mpro and PLpro) and RdRp for polyprotein processing and viral RNA synthesis [51]. Consequently, these conserved enzymes represent promising targets for broad-spectrum coronavirus therapeutics, whereas the more variable S protein primarily determines host tropism, receptor specificity, and immune escape.

High-resolution cryo-EM and X-ray crystallography have resolved SARS-CoV-2 spike (S) protein conformations in pre- and post-fusion states [52,53], enabling the design of entry inhibitors and vaccine antigens. Structural characterization of viral proteases Mpro and PLpro bound to inhibitors has similarly guided antiviral optimization, particularly through targeting active-site clefts to block replicase polyprotein processing. These insights underpin structure-guided drug design and mechanism-based inhibitor development.

Because structural characterization and antiviral development have been most extensively pursued for SARS-CoV-2, the following discussion focuses on COVID-19 as the best-characterized model for coronavirus antiviral drug discovery.

**Figure 2:**
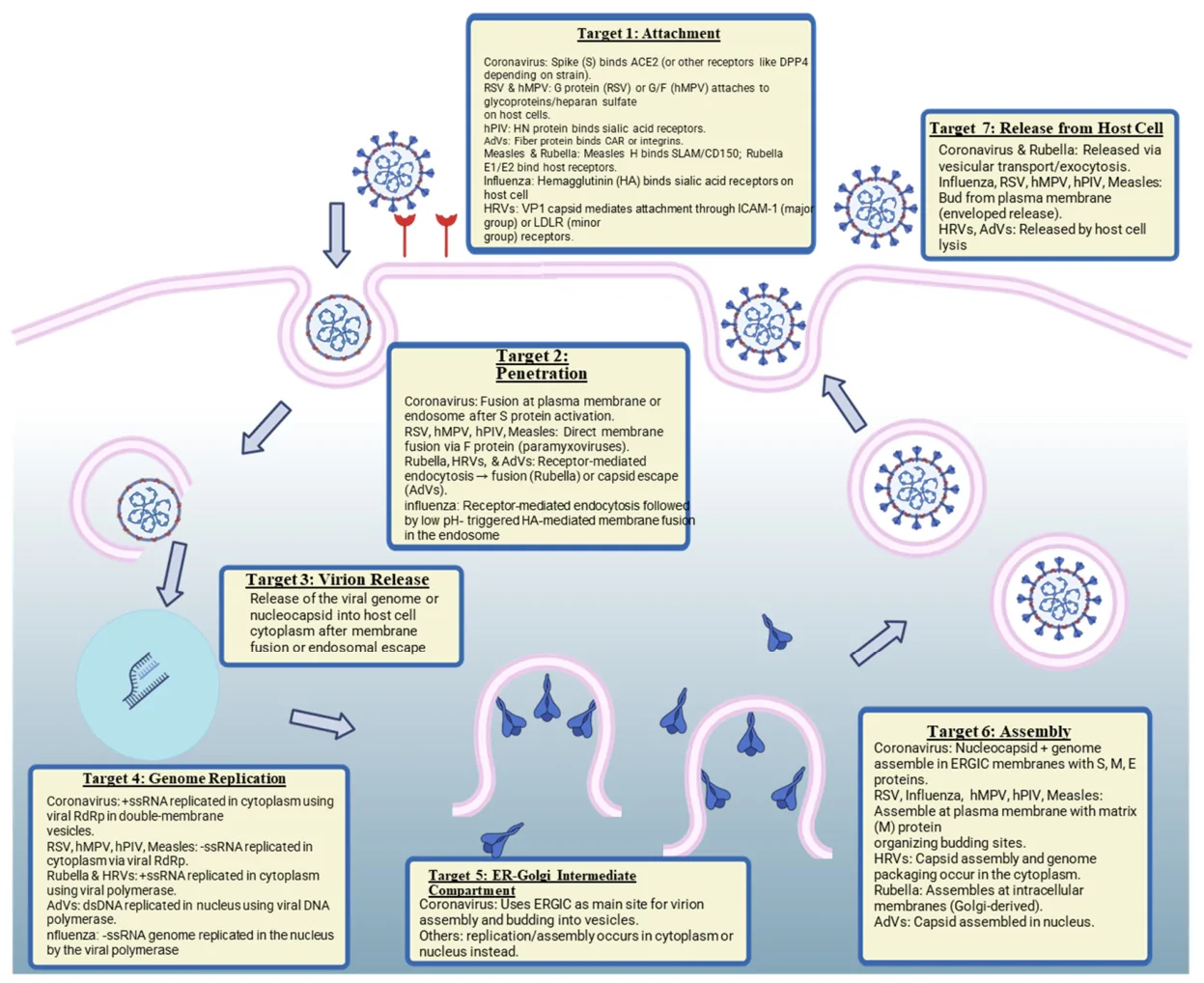
Viral replication cycle across different virus types. Viruses attach to host receptors, enter via membrane fusion or endocytosis, and release their genome; RNA viruses typically replicate in the cytoplasm using viral polymerases, while DNA viruses often utilize the nucleus and host machinery. Viral components are assembled into new virions and released through budding, exocytosis, or cell lysis depending on the virus type. Created in BioRender. Samrat, S. (2026) https://BioRender.com/n4s8dze.

SARS-CoV-2 entry is mediated by S protein binding to ACE2, followed by proteolytic activation by TMPRSS2 or endosomal cathepsin L (Figure 2) [54,55,56]. Cleavage at the S1–S2 boundary and S2′ site exposes fusion peptides that enable membrane fusion (Figure 2) [57,58]. Variations in S protein structure or cleavage efficiency can enhance transmissibility and immune evasion [59,60]. These mechanistic insights have informed updated vaccine designs, particularly stabilizing prefusion S conformations, and enabled development of entry inhibitors targeting ACE2–S interactions and host proteases [59].

**Figure 3:**
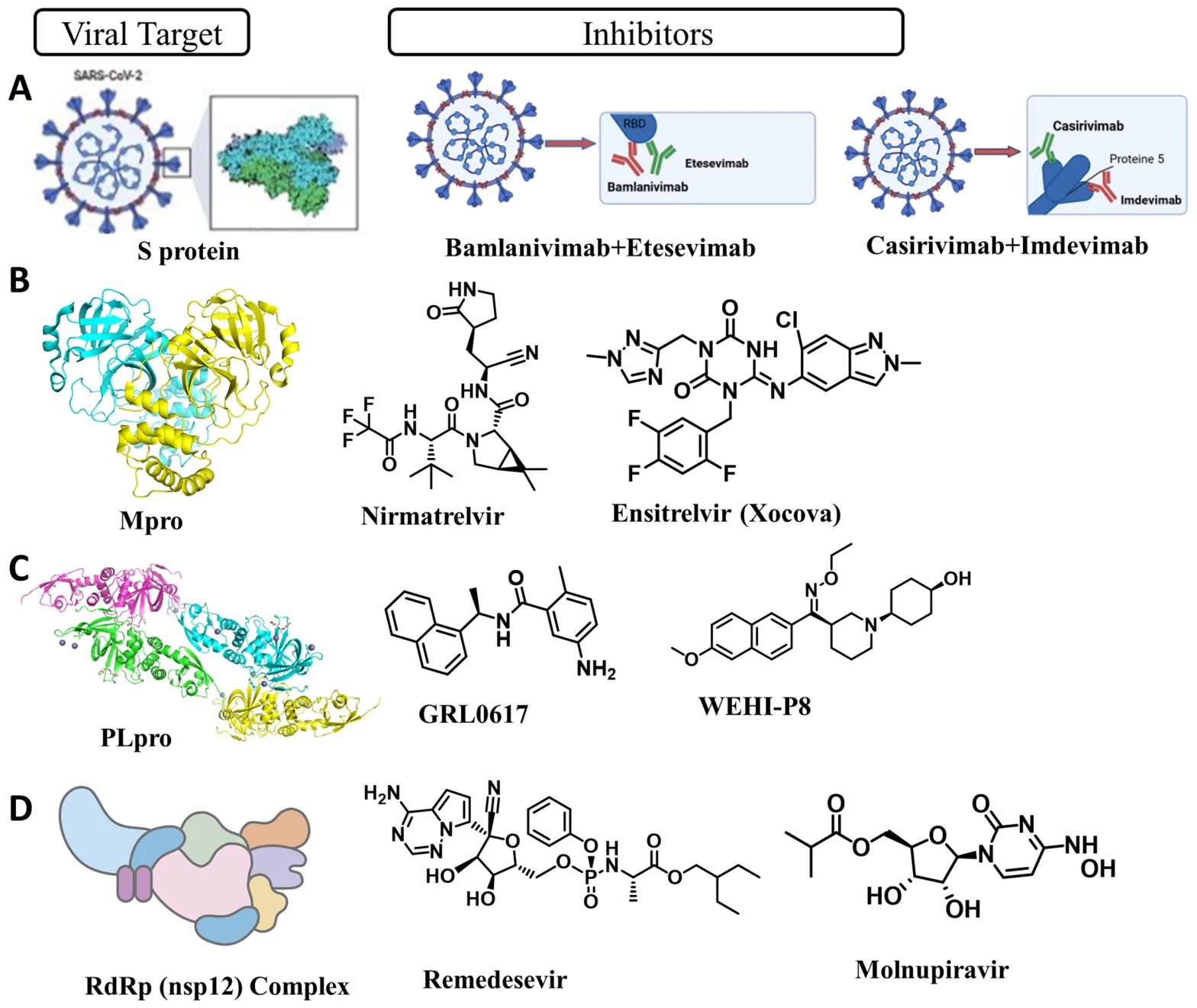
SARS-CoV-2 viral targets and mechanisms of antiviral inhibition. (**A**) Spike (S) protein inhibitors. Monoclonal antibodies (bamlanivimab + etesevimab and casirivimab + imdevimab) that bind the receptor-binding domain of the S protein, blocking ACE2 interaction and preventing viral entry. (**B**) Main protease (Mpro) inhibitors. Nirmatrelvir inhibits Mpro by forming a reversible covalent bond with its catalytic cysteine, blocking polyprotein cleavage and halting complex replication formation. Ensitrelvir (Xocova) inhibits SARS-CoV-2 Mpro by blocking proteolytic cleavage of viral polyproteins required for replication. Mpro crystal is modified from PDB 6XHU. (**C**) Papain-like protease (PLpro) inhibitors. GRL0617 and WEHI-P8 inhibit PLpro by occupying its substrate-binding site, preventing deubiquitinating activity, impairing viral processing, and restoring host antiviral immune signaling. PLpro crystal structure is modified from PDB 7CMD. (**D**) RNA-dependent RNA polymerase (RdRp) inhibitors. Remdesivir inhibits RdRp by acting as an adenosine nucleotide analog that causes delayed termination of viral RNA synthesis. Molnupiravir induces error catastrophe by introducing mutations into the viral genome during RNA replication. Created in BioRender. Samrat, S. (2026) https://BioRender.com/3ppvdpw (**A**) and tz1y0gd (**D** left panel). ChemDraw Desktop (version 26.0; Revvity Signals Software, Inc., Waltham, MA, USA) was used to prepare the chemical structures, and PyMOL (version 3.1.8; Schrödinger, LLC, New York, NY, USA) was used to generate and visualize the crystal structures.

COVID-19 therapeutics include direct-acting antivirals (DAAs), immunomodulators, and adjunctive treatments. DAAs target essential viral proteins such as RdRp, Mpro, PLpro, and the S protein, disrupting replication [61,62]. Remdesivir inhibits RdRp by acting as an adenosine nucleotide analog that causes delayed termination of viral RNA synthesis [63]; Molnupiravir, a nucleoside analog, inhibits RdRp by inducing error catastrophe through introducing mutations into the viral genome during RNA synthesis (Figure 3A) [64]. Because remdesivir competes with endogenous nucleotides during RNA synthesis, its antiviral activity depends on efficient intracellular conversion to the active triphosphate metabolite and sustained intracellular drug concentrations. Future optimization may therefore focus on improving oral bioavailability, tissue penetration, and resistance to viral proofreading mechanisms to enhance antiviral potency [65,66]. Nirmatrelvir targets Mpro by binding the catalytic active site and preventing cleavage of viral polyproteins required for replication (Figure 3B) [67]. The high degree of conservation within the Mpro catalytic site contributes to the broad antiviral potential of Mpro inhibitors. Ongoing efforts aim to improve binding affinity and maintain activity against resistance-associated substitutions arising within the protease active site [68]. Ensitrelvir (Xocova), another Mpro inhibitor, blocks viral polyprotein processing through inhibition of the main protease active site (Figure 3B), and became the first oral COVID-19 antiviral fully approved in Japan; additionally, it received FDA Fast Track designation and was recently approved by the U.S. FDA for post-exposure prevention of COVID-19 [69]. Structural optimization of next-generation Mpro inhibitors is directed toward enhancing potency, pharmacokinetic properties, and antiviral activity against newly emerging coronavirus variants [70]. Simnotrelvir and leritrelvir are orally administered SARS-CoV-2 main protease inhibitors that block proteolytic cleavage of viral polyproteins required for replication, thereby preventing the formation of the functional replication-transcription complex; both have received approval in China for the treatment of COVID-19 [71,72]. Further optimization of this inhibitor class is focused on increasing target affinity and preserving efficacy against mutations that alter the protease active site while maintaining oral bioavailability [73,74]. PLpro-targeting compounds such as GRL-0617 and WEHI-P8 inhibit protease activity by binding near the catalytic site and preventing viral polyprotein processing and deubiquitinating functions required for efficient replication and immune evasion (Figure 3C) [75,76]. In addition to suppressing viral polyprotein processing, PLpro inhibition has been shown to restore host antiviral signaling by preventing viral deubiquitinating and deISGylating activities [77]. Current efforts are focused on improving inhibitor selectivity and cellular permeability while minimizing off-target inhibition of host deubiquitinases.

In addition to DAAs, passive immunotherapies have been developed to target viral entry. The SARS-CoV-2 spike (S) protein is also targeted by neutralizing monoclonal antibody combinations including bamlanivimab plus etesevimab and casirivimab plus imdevimab, which bind the receptor-binding domain and block ACE2 interaction, thereby preventing viral attachment and entry into host cells (Figure 3D) [78]. Unlike DAAs, monoclonal antibodies function by extracellular neutralization of virions rather than inhibition of intracellular viral enzymes. Their clinical effectiveness is highly dependent on preservation of the targeted spike epitopes, making them particularly susceptible to loss of activity following antigenic drift [79]. Accordingly, the Emergency Use Authorizations for bamlanivimab plus etesevimab and casirivimab plus imdevimab were revoked after the emergence of Omicron-lineage variants, which acquired spike mutations that substantially reduced neutralization efficacy [80]. Future antibody development focuses on broadly neutralizing antibodies directed against conserved epitopes and multispecific antibody combinations capable of maintaining efficacy against newly emerging variants.

Among currently authorized therapies, Paxlovid (nirmatrelvir/ritonavir) and Veklury (remdesivir) have received FDA approval for the treatment of COVID-19 in eligible patients, while Lagevrio (molnupiravir) is available under Emergency Use Authorization (EUA) for certain adults with mild-to-moderate disease [81]. Immunomodulators (e.g., corticosteroids, IL-6 inhibitors) reduce hyperinflammation [82,83], while adjunctive therapies such as anticoagulants address complications like coagulopathy [84]. Emerging strategies include oligonucleotide-based therapies and next-generation vaccines. However, challenges remain, including antiviral resistance and heterogeneous patient responses.

Host-targeted antivirals (HTAs) represent an alternative approach by inhibiting host pathways required for viral replication, such as endosomal trafficking, protease activity, or immune signaling [85,86]. Because HTAs target conserved host processes rather than viral proteins, they generally impose a higher genetic barrier to resistance and may retain activity against newly emerging variants. Current efforts are focused on identifying host factors that are dispensable for normal cellular physiology but essential for viral replication [87]. While HTAs may offer broader activity and reduced resistance risk, their development is limited by potential toxicity and fewer late-stage clinical successes [85]. Avoiding disruption of essential host functions remains the primary barrier, though ongoing trials indicate continued potential.

**Table 3:** Comparison of key SARS-CoV-2 (coronavirus) viral targets based on sequence conservation, functional constraint, resistance rate, and broad-spectrum antiviral potential. It highlights that highly conserved and functionally essential enzymes like RdRp and Mpro show low resistance rates and strong broad-spectrum potential, whereas more variable proteins such as Spike and accessory proteins exhibit higher mutation tolerance and lower therapeutic robustness.

Viral Target	Sequence Conservation	Functional Constraint	Resistance Rate	Broad-Spectrum Potential	References
RdRp^i^	Very High	Essential catalytic enzyme; intolerant to mutations	Low	High	[88]
M^pro^	High	Required for polyprotein processing; conserved active site	Low–Moderate	High	[89,90]
PL^pro^	Moderate	Important but partially redundant; immune evasion roles	Moderate	Moderate	[91]
S	Low	Receptor binding tolerates mutations under immune pressure	High	Low	[92]
Accessory proteins	Very Low	Non-essential or host-specific functions	High	Very Low	[93]

### 3.2. Influenza Viruses

Human influenza viruses, of the family Orthomyxoviridae, possess segmented (~13.5 kb), negative-sense RNA genomes comprising eight gene segments that encode multiple structural and nonstructural proteins essential for viral entry, replication, assembly, and immune evasion (Figure 1) [94]. Recent structural studies have elucidated the architecture of key antiviral targets, including hemagglutinin (HA), neuraminidase (NA), the polymerase complex (PB1, PB2, and PA), and the matrix protein 2 (M2) ion channel. The polymerase complex and M2 are relatively conserved because their enzymatic functions are essential for viral replication, making them attractive targets for broad-spectrum antiviral development [95] (Table 4). In contrast, the surface glycoproteins HA and NA undergo continual antigenic drift under immune selection, requiring frequent vaccine reformulation and reducing susceptibility to immune-based therapies [96] (Table 4). High-value antiviral targets include the polymerase complex and NA, whose well-defined catalytic domains enable mechanism-based inhibition (Table 4). Surface-exposed HA is readily accessible for antibodies or entry inhibitors, whereas intracellular targets such as the polymerase complex and M2 require cell-permeable small molecules [97,98]. In terms of structural dynamics, HA undergoes extensive conformational rearrangements during membrane fusion, complicating vaccine and inhibitor design, while the polymerase complex and NA possess relatively rigid catalytic domains that facilitate structure-guided drug discovery [99,100].

High-resolution cryo-EM and X-ray crystallography have resolved multiple conformational states of HA, NA, and the heterotrimeric polymerase complex, enabling the rational design of entry inhibitors, neuraminidase inhibitors, and polymerase-directed antivirals [101,102,103]. Structural characterization of the PA endonuclease active site, PB2 cap-binding domain, and NA catalytic pocket bound to inhibitors has similarly guided antiviral optimization by defining key drug-binding interactions required for inhibition of viral transcription and progeny virus release. These structural advances continue to support structure-guided drug discovery and mechanism-based antiviral development.

Influenza virus entry is initiated by HA binding terminal sialic acid residues on host cell glycoproteins, followed by receptor-mediated endocytosis. Endosomal acidification activates the M2 proton channel, promoting viral uncoating and triggering HA-mediated membrane fusion (Figure 2) [104]. Variations in HA receptor-binding specificity, fusion stability, and NA enzymatic activity contribute to viral transmissibility, host adaptation, and immune escape [105]. These mechanistic insights have informed updated vaccine designs targeting stabilized HA conformations and facilitated development of antivirals directed against HA, NA, the polymerase complex, and M2.

**Figure 4:**
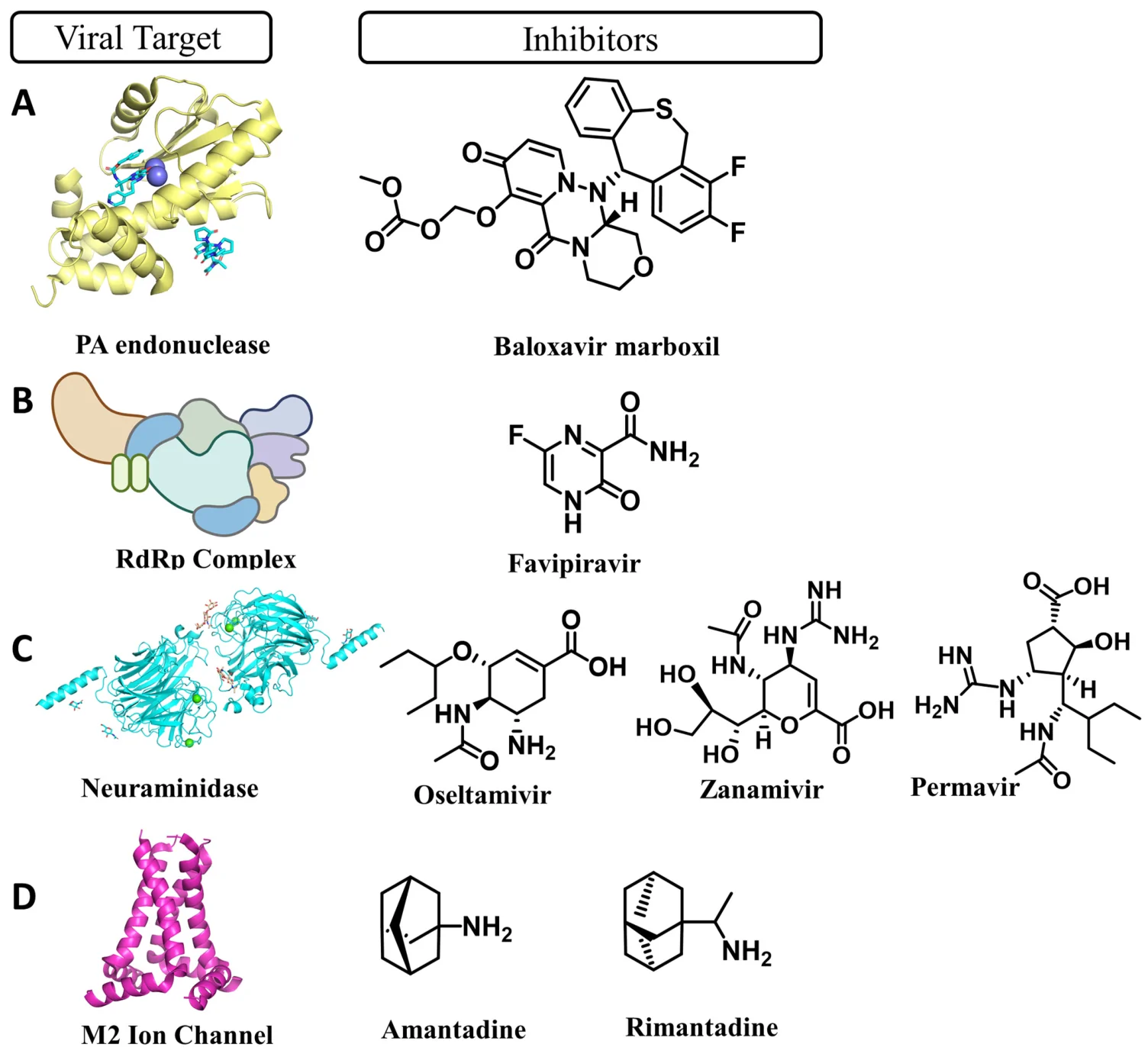
Influenza viral targets and mechanisms of antiviral inhibition. (**A**) PA endonuclease inhibitor. Baloxavir marboxil inhibits the viral PA endonuclease by blocking the cap-snatching process required for viral mRNA transcription, thereby preventing viral protein synthesis. PA endonuclease crystal is modified from PBD 7N68. (**B**) RNA-dependent RNA polymerase (RdRp) inhibitor. Favipiravir inhibits viral RdRp by acting as a purine nucleoside analog that is incorporated into viral RNA, inducing lethal mutagenesis and impairing viral genome replication. (**C**) Neuraminidase inhibitors. Oseltamivir, zanamivir, and peramivir inhibit neuraminidase by binding to its active site, preventing cleavage of sialic acid residues and blocking the release of newly formed virions from infected cells. The influenza neuraminidase protein crystal is modified from PBD 9GQX. (**D**) M2 ion channel inhibitors. Amantadine and rimantadine inhibit the viral M2 proton channel, preventing endosomal acidification required for viral uncoating and release of the viral genome into the host cell. M2 Ion Channel crystal is modified from PBD 2L0J. Created in BioRender. Samrat, S. (2026) https://BioRender.com/njwn37o (**B** left panel).

Influenza therapeutics include direct-acting antivirals (DAAs), supportive care, and adjunctive treatments. DAAs primarily target the viral polymerase complex, neuraminidase, and the M2 ion channel, thereby disrupting viral transcription, replication, and progeny virus release. Baloxavir marboxil inhibits the PA endonuclease by preventing cap-snatching, an essential step in viral mRNA synthesis (Figure 4A), while favipiravir acts as a nucleoside analog that is incorporated by the viral RNA polymerase, reducing replication fidelity and promoting lethal mutagenesis (Figure 4B) [106,107]. Because polymerase inhibitors target highly conserved enzymatic functions, they exhibit broad activity against influenza A and B viruses. However, resistance-associated substitutions within the PA endonuclease, particularly at residue I38, can reduce baloxavir susceptibility [108]. Current optimization efforts therefore focus on designing inhibitors that maintain high-affinity binding despite active-site substitutions and combining polymerase inhibitors with neuraminidase inhibitors to suppress resistance emergence [109]. Oseltamivir and zanamivir inhibit neuraminidase by binding its conserved catalytic pocket, preventing cleavage of terminal sialic acid residues and thereby blocking release of newly assembled virions (Figure 4C) [14]. Peramivir (Rapivab), an intravenously administered neuraminidase inhibitor, prevents progeny virion release by inhibiting the neuraminidase catalytic active site and is FDA-approved for the treatment of acute uncomplicated influenza (Figure 4C) [110,111]. Although neuraminidase inhibitors remain highly effective, substitutions such as H275Y reduce susceptibility to oseltamivir while preserving enzyme activity [112]. Consequently, next-generation neuraminidase inhibitors are being optimized to strengthen interactions with conserved catalytic residues outside common resistance hotspots and improve respiratory tissue retention. Adamantanes, a class of antiviral drugs that target the M2 ion channel of influenza A viruses, include Amantadine and rimantadine. They inhibit the M2 ion channel by blocking proton conductance required for viral uncoating (Figure 4D); however, widespread resistance among circulating influenza A viruses has largely eliminated their clinical utility [13,113]. Current efforts are therefore directed toward developing M2 inhibitors capable of retaining activity against resistant channel conformations.

In addition to DAAs, passive immunotherapeutic approaches targeting viral entry are under active investigation. Broadly neutralizing monoclonal antibodies directed against conserved epitopes within the HA stem inhibit the conformational changes required for membrane fusion, thereby preventing viral entry into host cells [114]. Unlike DAAs, these antibodies function through extracellular neutralization of virions rather than inhibition of intracellular viral enzymes. Because the HA stem is substantially more conserved than the immunodominant head domain, current development focuses on engineering broadly neutralizing antibodies with increased subtype breadth and prolonged serum half-life to improve protection against antigenically diverse influenza viruses.

Among currently approved therapies, oseltamivir, zanamivir, peramivir, and baloxavir marboxil are approved for treatment of influenza in appropriate clinical settings, whereas adamantanes are no longer recommended because of widespread antiviral resistance [13]. Early antiviral administration substantially improves clinical outcomes, although the narrow therapeutic window and continual antigenic evolution remain significant challenges. Emerging strategies include universal influenza vaccines targeting conserved HA stem epitopes, next-generation polymerase inhibitors, and combination antiviral therapies designed to reduce resistance development.

Host-targeted antivirals (HTAs) represent an alternative strategy by inhibiting host pathways required for influenza replication, including endosomal trafficking, host proteases involved in HA activation, and nuclear import of viral ribonucleoproteins [115,116]. Because HTAs target conserved host processes rather than viral proteins, they generally impose a higher genetic barrier to resistance and may retain activity against newly emerging influenza strains [23]. Current efforts are focused on identifying host factors that are dispensable for normal cellular physiology but essential for viral replication. While HTAs may provide broader antiviral activity and reduced resistance risk, their development remains limited by concerns regarding host toxicity and disruption of essential cellular functions [117].

**Table 4:** Comparison of key antiviral targets in human influenza viruses based on sequence conservation, functional constraints, resistance rates, and broad-spectrum antiviral potential. It shows that the highly conserved polymerase complex and neuraminidase provide the strongest cross-strain coverage, whereas the more variable hemagglutinin and M2 ion channel exhibit higher rates of resistance and reduced therapeutic durability.

Viral Target	Sequence Conservation	Functional Constraint	Resistance Rate	Broad-Spectrum/Strain Coverage Potential	References
Polymerase complex (PB1, PB2, PA)	Very High	Required for viral transcription and replication	Low–Moderate	High (conserved across influenza A and B viruses)	[95,118]
Neuraminidase (NA)	High	Required for progeny virion release	Moderate	High	[119,120]
Hemagglutinin (HA)	Moderate	Essential for viral attachment and membrane fusion	High	Moderate	[97,121]
M2 ion channel	Moderate–High	Required for endosomal acidification and viral uncoating	Very High	Moderate (Influenza A only)	[122,123]
Accessory/nonstructural proteins	Low	Immune evasion; not required for viral replication	High	Low	[124]

### 3.3. Respiratory Syncytial Virus (RSV)

Respiratory syncytial virus (RSV) is a ubiquitous negative-sense RNA virus in the family Pneumoviridae, with a genome approximately 15.2 kb in length, and is a leading cause of lower respiratory tract infections in infants and young children (Figure 1) [125,126].

The virus targets epithelial cells of the upper and lower respiratory tract [127,128]. RSV possesses two major surface proteins: the fusion (F) protein and the attachment (G) protein, which coordinate host cell binding and membrane fusion [129]. Engagement of these proteins enables viral penetration and promotes cell–cell fusion, resulting in the formation of multinucleated syncytia that are readily observed in vitro and are a hallmark of RSV infection in cell culture, although their extent and contribution to disease pathogenesis in vivo remain incompletely defined [129,130,131,132]. Following entry, the large (L) protein, which functions as the viral RNA-dependent RNA polymerase (RdRp), directs transcription and replication of the viral genome within the cytoplasm, making it necessary for viral propagation (Figure 2) [133,134]. The RSV nucleoprotein (N protein) encapsulates and protects the viral RNA genome while facilitating the formation of ribonucleoprotein complexes required for viral transcription and replication [135].

The fusion (F) protein is highly conserved, as it is critical for viral entry, whereas both F and the attachment (G) protein can mutate under antibody pressure, producing the fastest resistance (Table 5) [136,137,138]. The F protein and the N protein represent broad-spectrum targets, as their conserved sequence across RSV strains allows for vaccines or inhibitors to act widely (Table 5) [135,139,140]. High-value targets include F and N proteins, and the viral RNA polymerase (RdRp), which are essential for entry and replication, whereas the G protein is lower-value due to its variability and accessory immune-modulatory role [129,141]. Both F and G proteins are surface-exposed, making them accessible to neutralizing antibodies [129], while RdRp is intracellular and requires cell-permeable inhibitors [142]. The F protein’s metastable prefusion conformation presents a structural challenge, necessitating stabilization for effective therapeutics (Table 5) [134,139].

**Figure 5:**
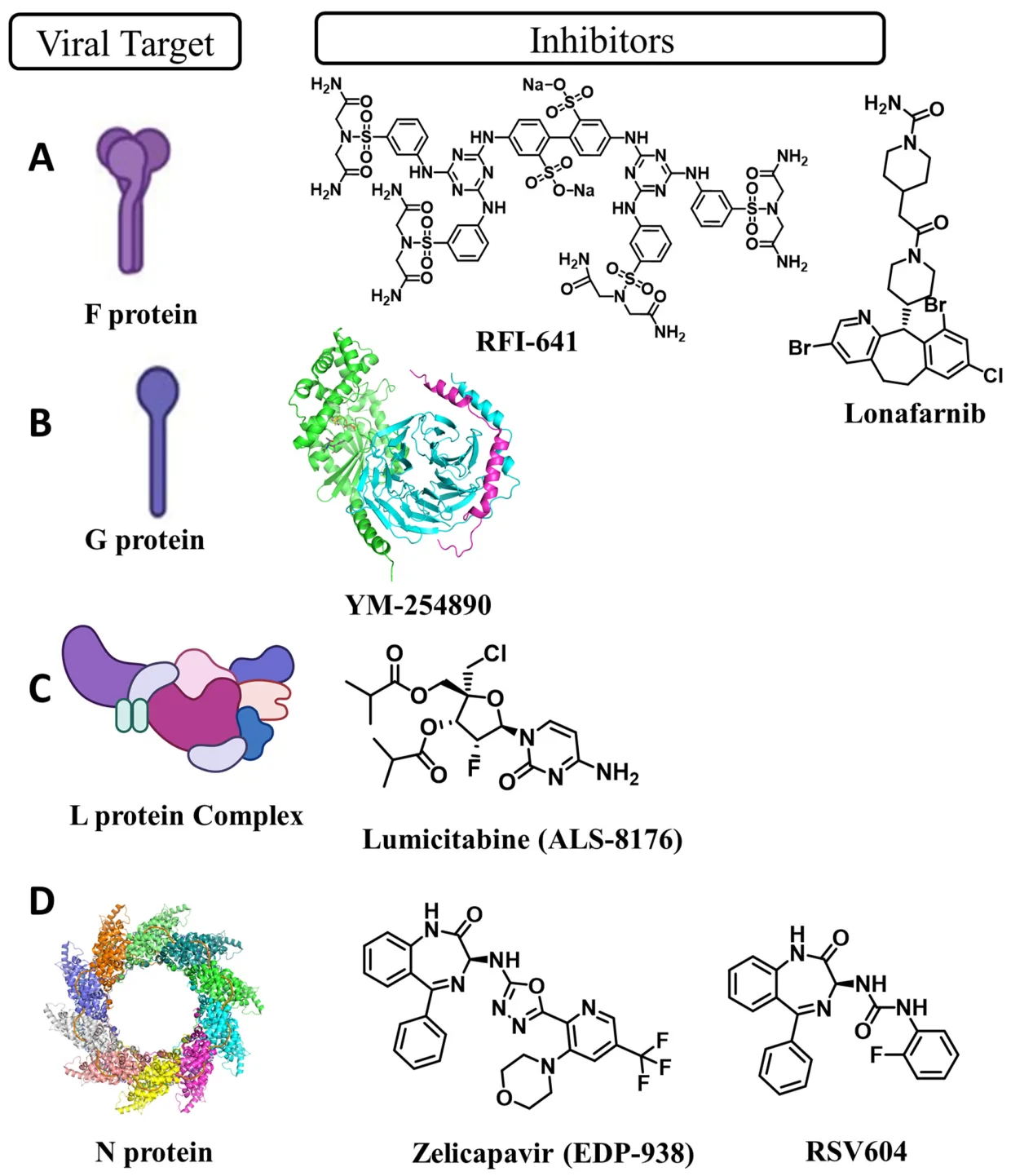
RSV and hMPV viral targets and mechanisms of antiviral inhibition. (**A**) Fusion (F) protein inhibitors. RFI-641 inhibits the F protein by preventing the conformational changes required for membrane fusion, while lonafarnib impairs F protein-mediated fusion through inhibition of host-dependent prenylation pathways. (**B**) Attachment (G) protein inhibitors. YM-254890 inhibits G protein-mediated viral attachment by blocking Gq protein signaling pathways involved in viral entry. (**C**) Large polymerase (L) protein inhibitors. Lumicitabine (ALS-8176) inhibits the viral RNA-dependent RNA polymerase (L protein) by acting as a nucleoside analog, disrupting viral RNA synthesis and replication. (**D**) Nucleoprotein (N) inhibitors. Zelicapavir (EDP-938) and RSV604 inhibit the N protein by disrupting ribonucleoprotein complex formation, impairing viral transcription and replication. N protein crystal is modified from PBD 2YHM. Created in BioRender. Samrat, S. (2026) https://BioRender.com/5gvemxd (**A** left panel), ky8wfj0 (**B** left panel) and 1iixuej (**C** left panel).

Though natural infection with RSV elicits both humoral and cellular immune responses, immunity is incomplete and wanes over time [143,144,145]. This occurrence has complicated vaccine development, and treatment of RSV infection remains largely supportive [145]. Ribavirin is a guanosine nucleoside analog that inhibits RSV replication by interfering with viral RNA polymerase activity, depleting intracellular GTP pools, and inducing mutations that impair viral RNA synthesis and protein production [146]. Because ribavirin exerts antiviral activity through multiple complementary mechanisms, including lethal mutagenesis and depletion of nucleotide pools, resistance has remained relatively uncommon despite decades of clinical use [147]. However, its routine use is limited by uncertain clinical benefits, toxicity concerns, difficult administration, and high cost [148]. In addition to direct-acting antivirals, passive immunotherapies have been developed to prevent RSV infection. Palivizumab is a monoclonal antibody that targets the RSV fusion (F) protein, preventing viral attachment and membrane fusion with host cells, thereby inhibiting viral entry and F protein-mediated cell–cell fusion [149]. Unlike DAAs, palivizumab functions through extracellular neutralization of virions by stabilizing the prefusion conformation of the F protein rather than inhibiting intracellular viral replication. The monoclonal antibody represented the first targeted prophylaxis, reducing hospitalization risk in high-risk infants but is limited by high cost, monthly dosing, and modest effectiveness [150,151]. Recent advances include nirsevimab (Beyfortus), a long-acting monoclonal antibody that binds a highly conserved prefusion epitope on the RSV fusion (F) protein, blocking membrane fusion and preventing viral entry into host cells [152]. Its extended half-life is achieved through Fc engineering that enhances neonatal Fc receptor (FcRn)-mediated recycling, enabling sustained serum concentrations and season-long protection following a single dose [153]. It has been FDA-approved for all infants entering their first RSV season, offering broad population-level protection with a single intramuscular dose [154]. Additional RSV-directed therapeutics target both the fusion (F) and attachment (G) proteins. RFI-641 is a small-molecule fusion inhibitor that binds the F protein and blocks the membrane fusion process required for viral entry and F protein-mediated cell–cell fusion, in which preclinical studies demonstrated potent antiviral activity against RSV (Figure 5A) [155]. By preventing the conformational rearrangements of the prefusion F protein that drive membrane merger, fusion inhibitors act early in the viral life cycle and limit both initial infection and F protein-mediated cell–cell spread, which has been extensively demonstrated in vitro [156]. Lonafarnib, a farnesyltransferase inhibitor originally developed for other indications, has also been shown to interfere with F protein-mediated fusion and reduce RSV replication in experimental models (Figure 5A) [157]. In contrast, YM-254890 targets host Gq-family G proteins involved in signaling pathways utilized during RSV infection, disrupting G protein-dependent viral processes and reducing viral propagation in vitro (Figure 5B) [158]. Because host-directed antivirals target cellular pathways rather than viral proteins, they may present a higher barrier to resistance, although maintaining selectivity and minimizing disruption of normal host signaling remain important considerations for clinical development.

The viral RNA-dependent RNA polymerase (L protein) and nucleoprotein (N protein) are significant antiviral targets as well. Lumicitabine (ALS-8176) inhibits L protein polymerase activity by inducing premature termination of viral RNA synthesis [159], while PC786 acts as an inhaled polymerase inhibitor that suppresses RSV replication (Figure 5C) [160]. The N protein has also emerged as a promising antiviral target, with Zelicapavir (EDP-938) disrupting nucleoprotein-dependent replication complex formation [161] and RSV604 interfering with nucleocapsid functions essential for viral transcription and replication (Figure 5D) [162]. Because nucleoprotein oligomerization and ribonucleoprotein complex assembly are highly conserved and indispensable for genome replication, N protein inhibitors may retain activity across diverse RSV strains.

Currently, ribavirin, palivizumab (Synagis), nirsevimab (Beyfortus), and recently approved clesrovimab (Enflonsia) for infants are the only FDA-approved RSV-directed therapeutics, whereas fusion inhibitors, polymerase inhibitors, and nucleoprotein inhibitors remain under clinical or preclinical investigation [163].

Despite persistent challenges in RSV antiviral development, advances in high-throughput screening and computational modeling have significantly accelerated RSV antiviral discovery by improving target identification, lead optimization, and mechanism-based drug design. For example, high-resolution structural characterization of the RSV prefusion F protein enabled the rational development of the monoclonal antibody nirsevimab, marking significant progress in RSV prevention and treatment [139].

### 3.4. Human Metapneumovirus (hMPV)

Human metapneumovirus (hMPV), a member of the *Paramyxoviridae* family and closely related to respiratory syncytial virus (RSV), is a negative-sense single-stranded RNA virus that causes respiratory illnesses, including bronchiolitis and pneumonia [164]. The hMPV genome is approximately 13 kb in length and encodes nine proteins (Figure 1) [165].

Of these, hMPV virions possess two key surface glycoproteins: the attachment protein (G) and the fusion protein (F). The G protein facilitates binding to host cell receptors, while the F protein mediates fusion of the viral envelope with the host cell membrane [166]. The F protein is highly conserved, as it is required for membrane fusion [167,168], whereas the G protein mutates under immune pressure, producing the fastest resistance (Table 5) [169,170]. Both F and the viral polymerase are broad-spectrum targets, as they are conserved across hMPV strains and amenable to inhibitors [171,172]. High-value targets include F and polymerase, which are essential for entry and replication, while the G protein is low-value due to its accessory role. The F protein is surface-exposed and accessible to antibodies or inhibitors [173], whereas the polymerase is intracellular and requires drugs that can cross cell membranes [174]. Additionally, the F protein’s prefusion conformation is metastable, complicating inhibitor or vaccine design (Table 5) [175].

Once inside the cytoplasm, the viral RNA-dependent RNA polymerase (RdRp) transcribes the genome, producing viral mRNA and replicating the viral RNA [176,177]. Viral particles are assembled in the cytoplasm and released through cell lysis [176,178]. The virus can also induce syncytia formation, contributing to tissue pathology, a phenomenon well documented in vitro; however, its frequency and contribution to tissue pathology during human infection remain less well established [165,179]. The virus’s pathogenesis involves evasion of host immune responses; for example, the G protein can inhibit innate immune signaling, allowing efficient viral replication and disease progression [180,181].

No HMPV-specific monoclonal antibodies have reached Phase 3 clinical trials, nor do any FDA-approved antiviral agents specifically target hMPV [182,183]. However, recent studies have identified several FDA-approved drugs that may be repurposed for HMPV treatment. Notably, in an in silico molecular docking study, remdesivir and peramivir were predicted to bind to the HMPV fusion (F) protein, with calculated binding affinities of −9.5 kcal/mol and −9.2 kcal/mol, respectively [183,184]. These computational findings provide preliminary support for drug repurposing but require experimental validation to confirm antiviral activity and therapeutic efficacy against hMPV.

To address the significant challenges that remain in hMPV antiviral development, including the absence of approved therapeutics and incomplete understanding of viral entry mechanisms, drug repurposing strategies have accelerated candidate discovery by leveraging existing safety and pharmacokinetic data. For instance, given the close phylogenetic relationship between RSV and hMPV, a recent computational drug-repurposing study identified the FDA-approved asthma drug prednisone as a potential hMPV therapeutic through its predicted interaction with Toll-like receptor 4 (TLR4) [185].

**Table 5:** Summary of key respiratory syncytial virus (RSV) and human metapneumovirus (hMPV) drug targets by comparing their sequence conservation, functional constraints, resistance rates, and breadth of antiviral coverage. It shows that highly conserved, replication-essential proteins like RdRp/L protein and the F fusion protein offer the strongest broad-spectrum potential, while variable attachment and accessory proteins are more prone to resistance and have limited therapeutic coverage.

Viral Target	Sequence Conservation	Functional Constraint	Resistance Rate	Broad-Spectrum/Strain Coverage Potential	References
F protein	High	Essential for viral entry and membrane fusion; intolerant to major structural changes	Moderate–High (mutates under antibody pressure)	High (conserved across RSV A and B strains)	[186]
RdRp/L protein	Very High	Required for transcription and replication of viral genome	Low	High	[187]
N protein	High	Essential for nucleocapsid formation, viral RNA encapsidation, and assembly of the transcription/replication complex; mutations are often poorly tolerated.	Low	High	[188]
G protein	Low	Accessory role in attachment and immune modulation; non-essential for replication	High	Low	[169,189]
Accessory proteins	Low–Moderate	Immune evasion; not strictly required for viral replication	High	Low	[186]

### 3.5. Parainfluenza Viruses (HPIVs)

Human parainfluenza viruses (HPIVs) are enveloped, negative-sense single-stranded RNA viruses belonging to the family *Paramyxoviridae*, genus Respirovirus (HPIV-1 and HPIV-3) and Rubulavirus (HPIV-2 and HPIV-4 [190]). The HPIV genome is approximately 15–19 kb in length, encoding six major structural proteins and several accessory proteins [191]. The two key surface glycoproteins are hemagglutinin neuraminidase (HN) and fusion protein (F), both critical to viral entry and pathogenesis. HN mediates attachment to host cells by binding sialic acid–containing glycoconjugates, while also possessing neuraminidase activity to facilitate virion release and prevent self-aggregation [191,192]. Following receptor binding, the F protein undergoes changes triggered by HN interaction, driving fusion of the viral envelope with the host cell membrane (Figure 2) [193,194].

From a therapeutic standpoint, the F protein is highly conserved and essential for viral entry [195], while HN mutates moderately under selective pressure, producing relatively fast resistance (Table 6) [196]. Both F and the viral polymerase are broad-spectrum targets, as they are functionally conserved across HPIV types (Table 6). High-value targets include F and polymerase, critical for entry and replication, whereas HN is considered low-value due to its bifunctional nature, which complicates drug targeting. The F protein is surface-exposed and accessible to neutralizing antibodies, whereas the polymerase is intracellular and requires cell-permeable inhibitors [197]. Additionally, the F protein undergoes significant conformational changes during prefusion-to-postfusion rearrangement, which must be considered in vaccine or inhibitor design (Table 6) [198,199].

Structural and computational studies have significantly advanced antiviral development against HPIV by elucidating the architecture and function of the hemagglutinin-neuraminidase (HN) and fusion (F) glycoproteins, and the integration of high-throughput screening has enabled the rapid identification and optimization of antiviral candidates with improved specificity and potency against HPIV. X-ray crystallography, for example, was used to resolve the structure of the HPIV hemagglutinin-neuraminidase (HN) protein in complex with receptor analogs and inhibitors, enabling the rational design of neuraminidase inhibitors such as BCX2798 and BCX2855 [200].

Despite decades of research, there are currently no FDA-approved antivirals for HPIV infection, and treatment remains largely supportive [201,202]. Nevertheless, several viral proteins present attractive opportunities for therapeutic intervention. Among these, the blocking of HPIV fusion (F) protein activation has been the most extensively studied. One of the most advanced candidates, DAS181 (fludase), is a recombinant sialidase that removes sialic acid receptors from the airway epithelium, thereby preventing viral attachment [203,204]. By targeting a host receptor rather than a viral protein, DAS181 is less susceptible to the emergence of resistance caused by viral mutations. Future optimization is focused on improving localized airway retention while minimizing desialylation of non-target respiratory tissues to preserve normal epithelial function.

**Figure 6:**
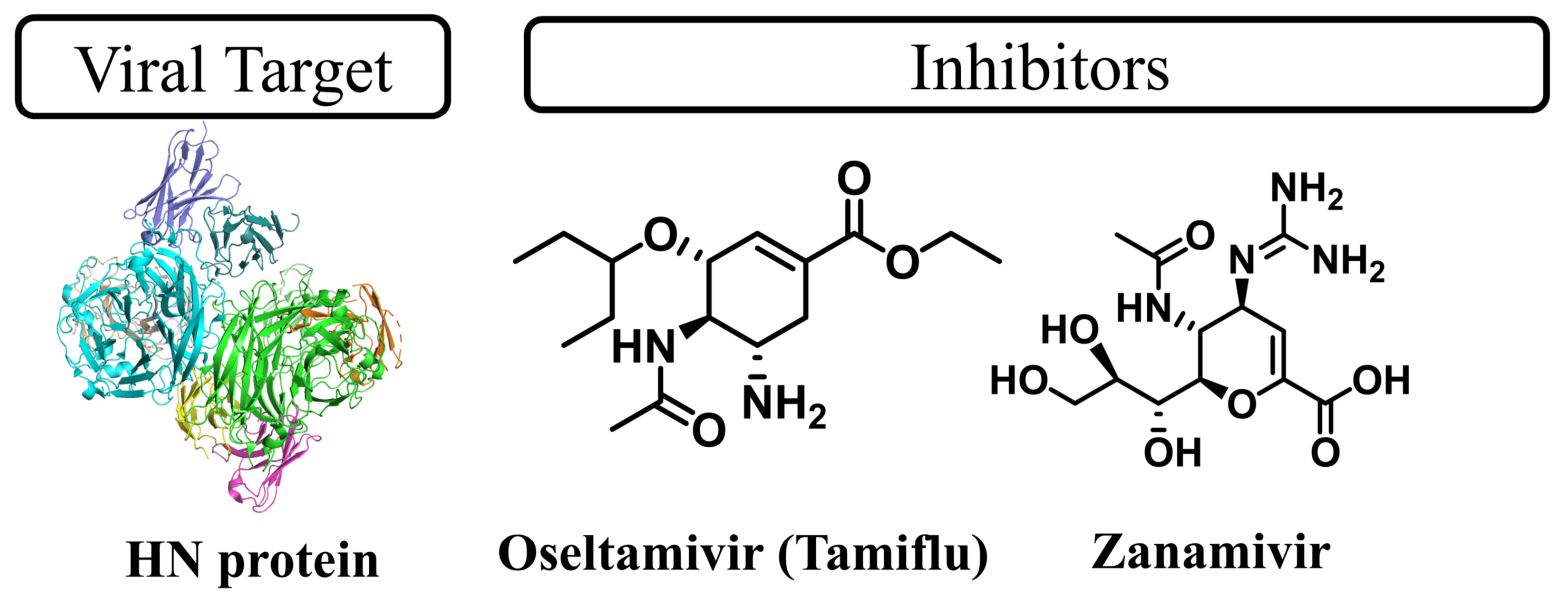
hPIV viral targets and mechanisms of antiviral inhibition. Oseltamivir and zanamivir inhibit Hemagglutinin-neuraminidase (HN) protein activity by binding the active site and blocking sialic acid cleavage, preventing viral release. HN protein crystal is modified from PDB 8TQI.

Additional therapeutic strategies have targeted the hemagglutinin-neuraminidase (HN) protein. Oseltamivir (Tamiflu), a neuraminidase inhibitor widely used against influenza viruses, has demonstrated inhibitory activity against HPIV HN protein function in experimental studies, reducing viral spread by blocking receptor cleavage and virion release (Figure 6) [200]. Similarly, zanamivir targets the HN neuraminidase active site, preventing sialic acid cleavage and impairing viral propagation (Figure 6) [205]. Because the HN protein coordinates receptor binding, neuraminidase activity, and activation of the fusion protein, its inhibition simultaneously disrupts multiple steps of the viral entry process. Structure-guided optimization of HN inhibitors is therefore directed toward increasing affinity for the conserved catalytic pocket while maintaining activity against sequence variation among HPIV serotypes. Although neither oseltamivir nor zanamivir is FDA-approved for HPIV infection, both compounds have served as important tools for investigating HN-directed antiviral strategies and the development of next-generation HPIV therapeutics.

The viral RNA polymerase complex, composed of the large (L) protein in association with phosphoprotein (P) and nucleoprotein (N), also represents a logical drug target. This complex directs genome replication and transcription, and inhibition at this stage could block viral propagation [206,207]. Because the L protein contains highly conserved catalytic domains that are indispensable for RNA synthesis, polymerase inhibitors may exhibit activity across multiple HPIV serotypes and potentially other members of the *Paramyxoviridae* family. Nucleoside analogues and other polymerase inhibitors have demonstrated activity in vitro, but most remain in preclinical stages or very early clinical testing [208]. Current efforts are focused on developing polymerase inhibitors with improved selectivity for the viral polymerase over host polymerases, enhanced intracellular activation of nucleoside analogs, and favorable pharmacokinetic properties that support effective drug concentrations within the respiratory epithelium.

**Table 6:** Summary of key human parainfluenza virus (hPIV) drug targets based on sequence conservation, functional constraint, resistance rate, and broad-spectrum antiviral potential. It highlights that highly conserved and essential enzymes like RdRp/L protein and the F fusion protein offer the strongest broad coverage across hPIV types, whereas more variable proteins such as HN and accessory proteins exhibit higher resistance risk and lower therapeutic robustness.

Viral Target	Sequence Conservation	Functional Constraint	Resistance Rate	Broad-Spectrum/Strain Coverage Potential	References
F protein	High	Essential for viral entry and membrane fusion; intolerant to major structural disruption; fusion occurs at neutral pH	Low–Moderate	High (conserved across HPIV types 1–4)	[195,209]
RdRp/L protein	Very High	Required for transcription and genome replication	Low	High	[210]
HN protein	Moderate	Bifunctional: mediates attachment and receptor-cleaving activity; complicates drug targeting	Moderate–High	Moderate–Low	[211]
Accessory proteins	Low–Moderate	Facilitate immune evasion or replication efficiency; not strictly required	High	Low	[212]

### 3.6. Measles & Rubella Viruses

Measles virus (MeV) is an enveloped, negative-sense single-stranded RNA virus belonging to the family *Paramyxoviridae*, genus *Morbillivirus* (Figure 1) [213]. The measles virus genome is approximately 15.9 kb in length and encodes six major structural proteins [213,214,215]. Viral entry is mediated by the hemagglutinin (H) protein, which binds the signaling lymphocyte activation molecule (SLAM/CD150) on immune cells [216,217,218] while the fusion (F) protein facilitates membrane fusion and viral penetration (Figure 2) [213]. Although measles and rubella are transmitted primarily through respiratory droplets and aerosols, they are not strictly classified as respiratory viruses because their primary pathogenesis is systemic rather than being confined to the respiratory tract. Following initial replication in the respiratory epithelium, both viruses disseminate throughout the body, causing multisystem disease. Rubella virus (RuV) is an enveloped, positive-sense single-stranded RNA virus of the *Matonaviridae* (previously *Togaviridae*) family and is the causative agent of rubella (German measles) [219,220,221]. The rubella virus genome is approximately 9.8 kb in length and encodes two nonstructural proteins (P150 and P90) involved in viral replication and three structural proteins [222]. The rubella virus envelope glycoproteins E1 and E2 enable viral entry into host cells, with E1 mediating membrane fusion and E2 supporting attachment, assembly, and proper glycoprotein function (Figure 2) [223]. Specifically, viral entry occurs via receptor-mediated endocytosis, followed by cytoplasmic replication, where viral RNA serves directly as mRNA for translation of viral proteins (Figure 2) [224,225,226].

The F protein in measles and the E1 protein in rubella is highly conserved and essential for viral entry [213,227,228,229], making them high-value targets. Surface glycoproteins such as H (MeV) and E1 (Rubella) are accessible to neutralizing antibodies but can mutate under immune pressure, resulting in fast resistance [229,230]. Viral polymerases are broadly conserved across these virus families, offering additional high-value, broad-spectrum targets, although they are intracellular and require cell-permeable inhibitors [231,232]. The F protein’s metastable prefusion conformation introduces challenges for inhibitor design, highlighting the need to consider protein dynamics in therapeutic development (Table 7) [233].

**Figure 7:**
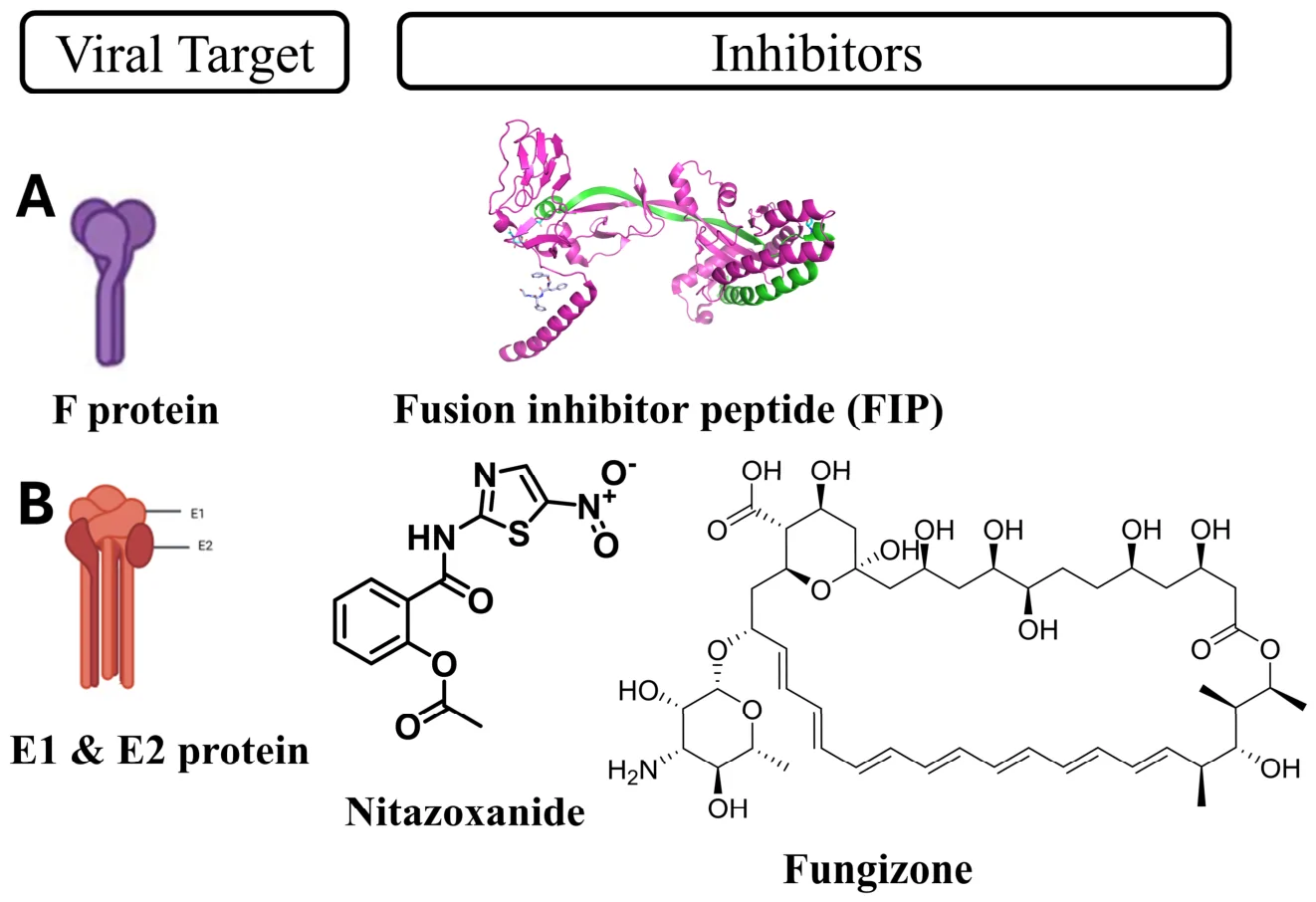
Measles and Rubell viral targets and mechanisms of antiviral inhibition. (**A**) Measles Fusion (F) protein inhibitor. Fusion inhibitor peptides (FIP) inhibit the F protein by binding to its heptad repeat regions, preventing the conformational changes required for membrane fusion and viral entry into host cells. FIP crystal is modified from PDB 5YZD. (**B**) Rubella envelope protein E1/E2 inhibitors. Nitazoxanide disrupts host-mediated glycoprotein maturation, impairing E1/E2 function, while amphotericin B (Fungizone) alters membrane integrity, reducing viral entry and fusion. Created in BioRender. Samrat, S. (2026) https://BioRender.com/5gvemxd (**A** left panel) and xr2a0ho (**B** left panel).

Although measles is preventable through the combined measles-mumps-rubella (MMR) vaccine [13], outbreaks continue to occur, primarily due to vaccine hesitancy or gaps in immunization coverage [234]. Currently, there are no FDA-approved antivirals for measles, though ribavirin has been studied in severe cases and immunocompromised patients, demonstrating limited efficacy. Ribavirin exerts antiviral activity through multiple complementary mechanisms, including inhibition of viral RNA synthesis, depletion of intracellular GTP pools, and induction of lethal mutagenesis, although these effects have not translated into consistent clinical benefit against measles virus. Fusion inhibitor peptide (FIP) derivatives inhibit measles virus infection by binding to the viral fusion (F) protein and preventing the membrane fusion events required for viral entry into host cells (Figure 7A); however, these peptides remain experimental and have not received FDA approval for clinical use [235]. By stabilizing the prefusion conformation of the F protein, these peptides prevent the structural rearrangements required for fusion of the viral and host cell membranes, thereby blocking infection at an early stage of the viral life cycle [236]. Future optimization is focused on increasing peptide stability, extending serum half-life, and improving delivery to the respiratory tract to enhance antiviral activity in vivo.

Like measles, rubella is preventable through the MMR vaccine [237], and there are no specific antiviral therapies available [237]. Several compounds have been investigated for their ability to disrupt the function of the rubella virus envelope glycoproteins E1 and E2. While nitazoxanide (NTZ), an FDA-approved drug for parasitic infections, has demonstrated broad-spectrum antiviral activity, including against rubella virus in laboratory settings, it is not approved for use against rubella in clinical practice (Figure 7B) [238]. Nitazoxanide (NTZ) inhibits rubella virus replication by disrupting host-mediated maturation and trafficking of the viral envelope glycoproteins E1 and E2, thereby impairing viral assembly and infectivity [238]. Because NTZ targets host pathways involved in viral protein maturation rather than viral enzymes directly, it may present a higher barrier to resistance than conventional direct-acting antivirals. Future development of NTZ-derived compounds is aimed at improving antiviral potency while preserving selectivity for host pathways that are preferentially exploited during viral infection. Another compound, amphotericin B (Fungizone), has been shown to inhibit rubella virus infection by altering membrane integrity and disrupting envelope-mediated fusion processes associated with E1 and E2 activity (Figure 7B) [239]. Further investigation is needed to identify derivatives that retain antiviral activity while minimizing the dose-limiting toxicity associated with amphotericin B. Although neither agent is approved for the treatment of rubella, both have provided valuable insights into E1/E2-directed antiviral strategies and potential host-targeted therapeutic approaches.

Given the absence of approved antivirals and the continued occurrence of outbreaks, alternative approaches such as drug repurposing, host-targeted therapies, and fusion protein-directed inhibitors are being investigated to expand treatment options. These efforts are increasingly supported by high-throughput screening and computational drug discovery platforms, which improve the speed and precision of antiviral candidate identification.

Overall, measles and rubella remain critical targets for immunization programs due to their high transmissibility and, in the case of rubella, severe congenital consequences. Vaccination remains the primary intervention, with research efforts continuing towards antiviral development.

**Table 7:** Comparison of key viral targets in measles and rubella based on sequence conservation, functional constraints, resistance rates, and broad-spectrum antiviral potential. It shows that highly conserved, replication-essential polymerases and fusion proteins (MeV F and Rubella E1) provide the strongest cross-strain coverage, while more variable surface attachment and accessory proteins exhibit higher resistance and lower therapeutic durability.

Viral Target	Sequence Conservation	Functional Constraint	Resistance Rate	Broad-Spectrum/Strain Coverage Potential	References
F protein (MeV)	High	Essential for viral entry and membrane fusion; metastable prefusion conformation	Moderate–High (mutates under immune pressure)	High	[240,241]
E1 protein (Rubella)	High	Required for viral entry and membrane fusion	Moderate–High	High	[223]
Viral polymerases (MeV & Rubella)	Very High	Required for viral transcription and replication	Low	High (conserved across strains)	[45,214]
Attachment glycoproteins (H protein- MeV)	Moderate	Mediates host receptor binding (SLAM/CD150); surface-exposed	High	Moderate	[242]
Accessory proteins	Low	Non-essential or redundant roles	High	Low	[243]

### 3.7. Rhinoviruses (HRVs)

Human rhinoviruses (HRVs), belonging to the family *Picornaviridae*, possess small (~7.2 kb), positive-sense single-stranded RNA genomes that encode a single polyprotein processed into structural and nonstructural proteins essential for viral entry, replication, and immune evasion (Figure 1) [244]. Recent structural studies have elucidated the architecture of key antiviral targets, including the capsid protein VP1, the 3C protease (3Cpro), the RNA-dependent RNA polymerase (3Dpol), and host cell receptors involved in viral attachment. The 3C protease and 3D polymerase are highly conserved because their enzymatic functions are essential for viral replication, making them attractive targets for broad-spectrum antiviral development (Table 8) [245]. In contrast, the surface-exposed capsid proteins exhibit substantial sequence diversity across more than 160 rhinovirus serotypes, limiting the durability of capsid-directed therapies and vaccine development (Table 8) [246]. High-value antiviral targets include 3Cpro and 3Dpol, whose conserved catalytic domains enable mechanism-based inhibition (Table 8) [245,247,248]. Surface-exposed VP1 is readily accessible for entry inhibitors, whereas intracellular enzymes such as 3Cpro and 3Dpol require cell-permeable small molecules [249]. In terms of structural dynamics, the viral capsid undergoes conformational rearrangements during receptor binding and uncoating, complicating the design of broadly active entry inhibitors, while the protease and polymerase possess relatively rigid catalytic sites that facilitate structure-guided drug discovery (Table 8) [250,251].

High-resolution X-ray crystallography and cryo-EM have resolved multiple conformational states of the rhinovirus capsid, 3C protease, and RNA-dependent RNA polymerase, enabling the rational design of capsid-binding compounds and enzyme-directed antivirals [250,252]. Structural characterization of VP1 hydrophobic pockets and the catalytic sites of 3Cpro and 3Dpol bound to inhibitors have similarly guided antiviral optimization by defining key drug-binding interactions required for inhibition of viral entry and replication [249,253]. These structural advances continue to support structure-guided drug discovery and mechanism-based antiviral development.

Rhinovirus entry is initiated by binding of the viral capsid to host receptors, most commonly intercellular adhesion molecule-1 (ICAM-1) for major-group rhinoviruses and low-density lipoprotein receptor (LDLR) family members for minor-group viruses, followed by receptor-mediated endocytosis [254]. Receptor engagement induces conformational changes within the capsid that promote viral uncoating and release of the viral RNA genome into the cytoplasm (Figure 2). Variations in receptor usage and capsid structure contribute to host tropism, transmission, and immune evasion [255]. These mechanistic insights have informed the development of capsid-binding antivirals and receptor-targeted strategies designed to prevent viral attachment and entry.

**Figure 8:**
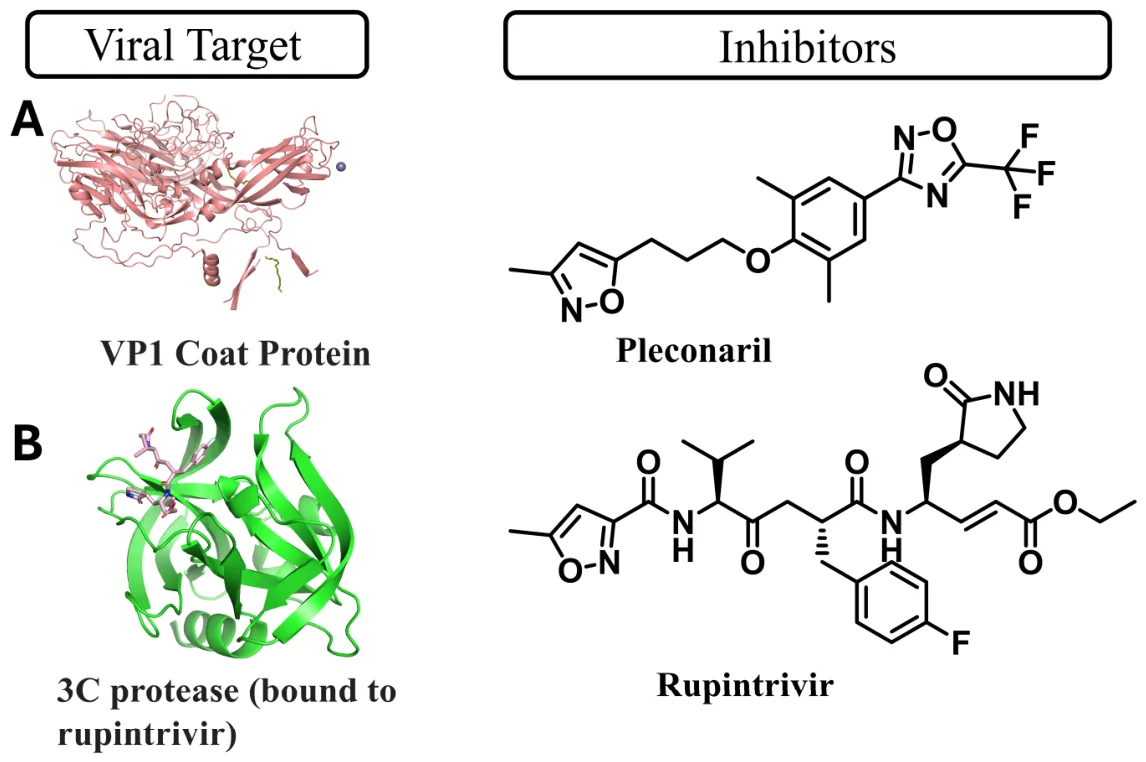
Rhinovirus viral targets and mechanisms of antiviral inhibition. (**A**) VP1 coat protein inhibitor. Pleconaril inhibits the VP1 coat protein by binding to a hydrophobic pocket within the capsid, stabilizing its structure and preventing receptor binding, viral uncoating, and genome release. VP1 coat protein crystal is modified from PBD 1AYM. (**B**) 3C Protease inhibitor. Rupintrivir inhibits the viral 3C protease by binding to its catalytic active site, preventing cleavage of the viral polyprotein into functional proteins required for viral replication. 3C protease crystal is modified from PBD 6KU8.

Rhinovirus therapeutics remain largely investigational, with direct-acting antivirals primarily targeting the viral capsid, 3C protease, and RNA-dependent RNA polymerase. Pleconaril binds a hydrophobic pocket within VP1, stabilizing the viral capsid and preventing the conformational changes required for receptor attachment and uncoating (Figure 8A) [256]. Although pleconaril demonstrated antiviral activity in clinical trials, variable efficacy across rhinovirus serotypes and drug interactions limited its clinical approval [257,258]. Current optimization efforts focus on developing capsid inhibitors that retain activity across diverse serotypes by targeting highly conserved structural features of the VP1 pocket while improving pharmacokinetic properties. Rupintrivir inhibits 3C protease by binding the catalytic active site and preventing cleavage of the viral polyprotein required for formation of the replication complex (Figure 8B) [6,253]. Because the catalytic residues of 3Cpro are highly conserved among picornaviruses, protease inhibitors possess broad-spectrum antiviral potential. However, limited bioavailability and suboptimal clinical efficacy have restricted their development [259]. Current efforts therefore focus on improving oral bioavailability, increasing intracellular drug exposure, and enhancing binding interactions with conserved residues surrounding the catalytic site to maintain activity against genetically diverse rhinoviruses.

In addition to DAAs, host-targeted strategies have been investigated to inhibit viral attachment and replication. Soluble ICAM-1 derivatives and receptor-blocking compounds prevent viral binding to host cells by competitively inhibiting interactions between the viral capsid and cellular receptors [260,261]. Unlike capsid-binding antivirals, these approaches target conserved host factors rather than viral proteins and may therefore reduce the likelihood of resistance development. However, their clinical application remains limited by incomplete coverage of all rhinovirus receptor groups and challenges associated with efficient delivery to the respiratory epithelium [262]. Currently, no antiviral therapies have received FDA approval for the treatment of rhinovirus infection, and clinical management remains primarily supportive [13]. The extensive antigenic diversity of rhinoviruses, together with the large number of circulating serotypes, has hindered both antiviral and vaccine development. Emerging strategies therefore focus on broad-spectrum inhibitors directed against conserved viral enzymes, next-generation capsid inhibitors with improved serotype coverage, and host-targeted approaches capable of limiting viral entry while minimizing toxicity.

Host-targeted antivirals (HTAs) represent an alternative strategy by inhibiting host pathways required for rhinovirus replication, including receptor-mediated viral attachment, endosomal trafficking, and intracellular signaling pathways that support viral replication [263,264]. Because HTAs target conserved host processes rather than highly variable viral capsid proteins, they generally impose a higher genetic barrier to resistance and may retain activity against numerous rhinovirus serotypes. Current efforts are focused on identifying host factors that are dispensable for normal cellular physiology but essential for viral replication [264]. While HTAs may provide broader antiviral activity and reduced resistance risk, their development remains limited by concerns regarding host toxicity and disruption of essential cellular functions.

**Table 8:** Comparison of key antiviral targets in human rhinoviruses based on sequence conservation, functional constraints, resistance rates, and broad-spectrum antiviral potential. It shows that the highly conserved 3C protease and RNA-dependent RNA polymerase provide the strongest cross-serotype coverage, whereas the structurally diverse capsid protein VP1 exhibits higher resistance potential and reduces therapeutic durability.

Viral Target	Sequence Conservation	Functional Constraint	Resistance Rate	Broad-Spectrum/Strain Coverage Potential	References
3C protease (3Cpro)	Very High	Required for viral polyprotein processing	Low	High	[245,265]
RNA-dependent RNA polymerase (3Dpol)	Very High	Required for viral RNA transcription and replication	Low	High	[248,251]
Capsid protein VP1	Moderate	Required for viral attachment and uncoating	High	Moderate	[266,267]
Host receptors (ICAM-1/LDLR)	High	Required for viral attachment and entry	Low	High	[252,268]
Accessory/nonstructural proteins	Low	Immune evasion and host interactions	High	Low	[262]

### 3.8. Adenoviruses (AdVs)

Adenoviruses (AdVs) are non-enveloped, double-stranded DNA viruses belonging to the family Adenoviridae (Figure 1). They possess an icosahedral capsid and a genome approximately 30–40 kilobases in length, encoding over 40 proteins [269,270]. There are more than 50 human serotypes classified into seven species (A-G), with AdV types 1, 2, 3, 4, and 5 being most commonly associated with human disease [270,271]. Clinically, they cause a range of illnesses, including upper and lower respiratory tract infections, conjunctivitis, gastroenteritis, and cystitis [272]. AdV replication occurs exclusively in the host cell nucleus [273]. The viral lifecycle begins with the attachment of the fiber protein to the coxsackievirus and adenovirus receptor (CAR) on the host cell surface (Figure 2) [273,274,275]. This binding facilitates clathrin-mediated endocytosis, leading to the internalization of the virus [273,276,277].

The DNA polymerase is highly conserved and essential for viral genome replication, making it a high-value target (Table 9) [278,279]. In contrast, early regulatory proteins such as E1A and E2A, as well as fiber protein, are lower-value targets due to variability or complex cellular interactions [280]. The fiber protein is surface-exposed and accessible to neutralizing antibodies, whereas DNA polymerase is intracellular and requires cell-permeable inhibitors [270,281]. Broad-spectrum strategies could target the polymerase or host factors critical for replication across multiple adenovirus serotypes (Table 9). Compared to paramyxoviruses, adenovirus capsids are structurally stable, simplifying drug targeting relative to viruses with dynamic fusion proteins [282,283].

**Figure 9:**
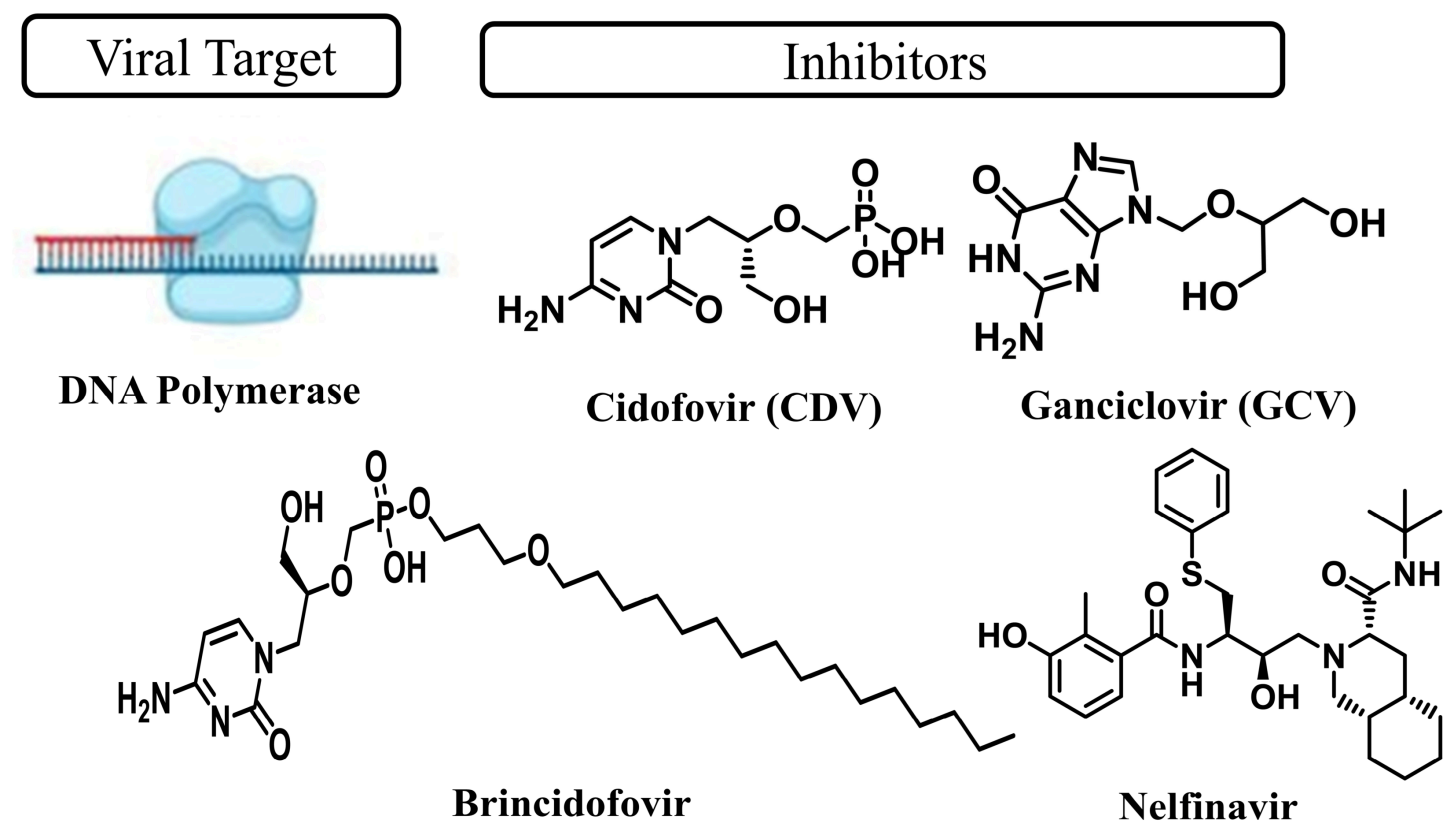
ADVs viral targets and mechanisms of antiviral inhibition. Cidofovir and ganciclovir act as nucleoside analogs that inhibit DNA polymerase through competitive incorporation and chain termination. Brincidofovir inhibits viral DNA polymerase by acting as a lipid-conjugated nucleotide analog that disrupts viral DNA synthesis, while nelfinavir indirectly impairs DNA polymerase-dependent replication by inhibiting viral protein processing and reducing the formation of functional replication complexes. Created in BioRender. Samrat, S. (2026) https://BioRender.com/6y0pr0c (DNA Polymerage).

No FDA-approved antiviral agents specifically target adenoviruses (AdVs) [284]. In immunocompromised patients, some antivirals have been used off-label with varying success. Cidofovir, a nucleotide analogue that inhibits viral DNA polymerase, is the most commonly used and has shown efficacy in hematopoietic stem cell transplant patients, though its use is limited by nephrotoxicity (Figure 9) [285,286,287]. Following intracellular phosphorylation to its active diphosphate form, cidofovir competes with natural deoxycytidine triphosphate during viral DNA synthesis, resulting in premature termination or marked slowing of genome elongation [288]. Current efforts to improve cidofovir-based therapies focus on reducing renal accumulation through targeted prodrug design while maintaining high intracellular concentrations within infected tissues. Ganciclovir is a guanosine nucleoside analog that inhibits adenoviral DNA polymerase, disrupting viral DNA synthesis and preventing genome replication [289]. Its antiviral activity likewise depends on intracellular conversion to the active triphosphate metabolite, allowing competitive inhibition of viral DNA polymerase during genome replication. It has also been used off-label, but its effectiveness against AdVs is less established (Figure 9) [289]. Brincidofovir, a lipid-conjugated prodrug of cidofovir that is more efficiently taken up by cells and then converted to cidofovir intracellularly, showed preclinical activity (Figure 9) [290,291]. Lipid conjugation substantially improves oral bioavailability and cellular uptake while reducing the renal toxicity associated with cidofovir [292]. Continued optimization of lipid-conjugated nucleotide analogs is directed toward improving intracellular drug delivery and limiting dose-limiting gastrointestinal toxicity.

Investigational agents like nelfinavir, which suppresses adenoviral propagation by inhibiting the lytic cell-free transmission of virions and limiting viral spread between host cells, have demonstrated in vitro activity (Figure 9) [293]. By limiting extracellular dissemination rather than directly inhibiting viral genome replication, nelfinavir may complement polymerase inhibitors in combination antiviral strategies.

Efforts to develop therapeutics focus on both viral and host factors critical for replication, including viral proteins like E1A and E2A, though no therapies targeting these proteins have reached clinical use. Because E1A functions as a master transcriptional regulator that initiates viral gene expression and E2A is essential for viral DNA replication, inhibition of these early proteins could suppress multiple downstream stages of the adenoviral life cycle. Adenoviruses exploit host pathways, including HSP90 for protein maturation, mTOR for protein synthesis, and PTKs for infection [294,295,296]. These factors are also promising antiviral targets, as inhibitors (e.g., 17-AAG, mTOR, and PTK inhibitors) reduce replication in vitro [297]. Host-directed therapies may provide a higher genetic barrier to resistance than direct-acting antivirals because they target conserved cellular pathways rather than rapidly evolving viral proteins. Current optimization efforts are focused on selectively disrupting host factors that are preferentially utilized during adenoviral infection while minimizing toxicity associated with inhibition of essential cellular signaling pathways.

Though antiviral agents approved for clinical use to treat adenoviruses are lacking, the integration of high-throughput screening, rational drug design, and artificial intelligence-based approaches is already accelerating adenovirus antiviral discovery by enabling the rapid identification and optimization of compounds targeting both viral proteins and host-dependency factors. Furthermore, advances in structural biology, including cryo-electron microscopy and high-resolution protein modeling, are improving the characterization of adenoviral capsid proteins and virus–host interactions, facilitating the development of more selective and effective antiviral candidates for potential clinical use [298].

**Table 9:** Outlines of key adenovirus (AdV) antiviral targets by comparing sequence conservation, functional constraints, resistance rates, and broad-spectrum coverage potential. It shows that highly conserved, replication-essential components such as DNA polymerase and structural capsid proteins provide the strongest therapeutic targets, while variable surface and regulatory proteins like fiber and early accessory factors exhibit higher resistance and limited cross-serotype coverage.

Viral Target	Sequence Conservation	Functional Constraint	Resistance Rate	Broad-Spectrum/Strain Coverage Potential	References
DNA polymerase	Very High	Essential for viral genome replication	Low	High (conserved across AdV serotypes)	[299,300]
Fusion/capsid proteins (e.g., penton, hexon)	High	Structural integrity is critical for viral assembly and infection	Low–Moderate	Moderate–High (structurally conserved across serotypes)	[301,302]
Fiber protein	Low–Moderate	Mediates attachment to host CAR receptor; surface-exposed	Moderate–High	Low–Moderate (variable among serotypes)	[303]
Early regulatory proteins (E1A, E2A)	Low	Modulate host machinery; not strictly essential for replication in all contexts	High	Low	[304]
Accessory Proteins	Low–Moderate	Facilitate viral replication but not strictly required	High	Low	[299]

## 4. Challenges and Future Directions

Despite substantial advances in target-based antiviral development, significant therapeutic gaps remain across human respiratory viruses. While approved antivirals are available for SARS-CoV-2, influenza virus, and RSV, effective therapies remain unavailable for seasonal human coronaviruses, rhinoviruses, human metapneumovirus, parainfluenza viruses, adenoviruses, measles, and rubella. Viral diversity, rapid mutation, and the emergence of antiviral resistance continue to limit the durability of many existing therapeutics, particularly those directed against highly variable surface glycoproteins. Future antiviral development should therefore increasingly prioritize highly conserved viral proteins, including RNA-dependent RNA polymerases, viral proteases, and fusion proteins, whose essential functions constrain sequence variation and make them attractive targets for broad-spectrum antiviral agents. Such therapies could provide activity against multiple viral species while strengthening preparedness for newly emerging respiratory pathogens.

Combination antiviral therapy represents another promising strategy for improving clinical outcomes and limiting antiviral resistance. Similar to treatment paradigms established for HIV and hepatitis C virus, combining agents that target distinct stages of the viral life cycle, such as entry, polyprotein processing, and genome replication, may produce synergistic antiviral activity while reducing the likelihood that resistance-conferring mutations arise against any single therapeutic target. Pairing direct-acting antivirals with host-targeted therapies may further increase the genetic barrier to resistance by simultaneously inhibiting viral proteins and host pathways required for replication. Although combination regimens have demonstrated clinical benefit during the COVID-19 pandemic, their application to other respiratory viruses remains largely unexplored and represents an important area for future investigation.

Recent advances in structural biology and artificial intelligence (AI) are also transforming antiviral drug discovery. High-resolution cryo-electron microscopy and X-ray crystallography have enabled atomic-level characterization of viral proteins, facilitating rational inhibitor design against conserved active sites. Complementing these experimental approaches, AI-driven protein structure prediction, virtual screening, molecular dynamics simulations, and machine learning algorithms are accelerating target identification, lead optimization, and prediction of resistance-associated mutations. In parallel, AI-assisted drug repurposing has expanded opportunities to rapidly identify existing therapeutics with antiviral activity, reducing both development time and cost. Continued integration of computational approaches with structural biology and experimental virology is expected to accelerate the development of next-generation antiviral therapeutics with improved potency, broader spectrum of activity, and greater resilience against viral evolution. Collectively, the studies highlighted in this review demonstrate that understanding conserved viral targets and their structural and functional properties provides the foundation for rational antiviral design, offering a framework for the development of more effective therapies against both endemic and emerging respiratory viruses.

## Figures and Tables

**Table 1:** Registered and approved antiviral therapies for human respiratory viruses. It summarizes currently registered or authorized antiviral agents and monoclonal antibodies together with their associated respiratory viruses and regulatory approval status.

Drug	Virus(es)	Regulatory Status/Approval
Remdesivir (Veklury)	SARS-CoV-2	FDA approved
Nirmatrelvir/ritonavir (Paxlovid)	SARS-CoV-2	FDA approved
Molnupiravir (Lagevrio)	SARS-CoV-2	FDA Emergency Use Authorization (U.S.); approved in several countries
Ensitrelvir (Xocova)	SARS-CoV-2	Fully approved in Japan; FDA approved for post-exposure prevention
Simnotrelvir	SARS-CoV-2	Approved in China
Leritrelvir	SARS-CoV-2	Approved in China
Palivizumab (Synagis)	RSV	FDA approved (high-risk infants)
Nirsevimab (Beyfortus)	RSV	FDA approved
Clesrovimab (Enflonsia)	RSV	FDA approved
Ribavirin	RSV	FDA approved
Oseltamivir (Tamiflu)	Influenza A & B	FDA approved
Zanamivir (Relenza)	Influenza A & B	FDA approved
Peramivir (Rapivab)	Influenza A & B	FDA approved
Baloxavir marboxil (Xofluza)	Influenza A & B	FDA approved
Amantadine	Influenza A	FDA approved, but no longer recommended because of widespread resistance
Rimantadine	Influenza A	FDA approved, but no longer recommended because of widespread resistance

**Table 2:** Promising investigation and repurposed compounds targeting human respiratory viruses. It summarizes representative investigational and repurposed antiviral compounds discussed throughout this review together with their associated respiratory viruses and current stage of development. The table highlights the diverse therapeutic strategies under investigation, including novel direct-acting antivirals, host-targeted approaches, and drug repurposing efforts aimed at expanding treatment options for respiratory viral infections.

Compound	Virus(es)	Current Development Stage/Status
GRL-0617	SARS-CoV-2	Preclinical
WEHI-P8	SARS-CoV-2	Preclinical
Bamlanivimab + Etesevimab	SARS-CoV-2	Former FDA Emergency Use Authorization (revoked)
Casirivimab + Imdevimab	SARS-CoV-2	Former FDA Emergency Use Authorization (revoked)
RFI-641	RSV	Preclinical
Lonafarnib	RSV	Preclinical
YM-254890	RSV	Preclinical
Lumicitabine (ALS-8176)	RSV	Clinical development discontinued (completed Phase II)
PC786	RSV	Phase II
Zelicapavir (EDP-938)	RSV	Phase II
RSV604	RSV	Phase II
Remdesivir *	hMPV	In silico repurposing candidate
Peramivir *	hMPV	In silico repurposing candidate
Prednisone *	hMPV	In silico repurposing candidate
DAS181 (Fludase)	HPIV	Phase III
Oseltamivir *	HPIV	Experimental repurposing candidate
Zanamivir *	HPIV	Experimental repurposing candidate
Experimental polymerase inhibitors	HPIV	Preclinical
Cidofovir *	Adenovirus	Off-label clinical use
Ganciclovir *	Adenovirus	Off-label clinical use
Brincidofovir	Adenovirus	Clinical development
Nelfinavir	Adenovirus	Preclinical
17-AAG	Adenovirus	Preclinical
mTOR inhibitors	Adenovirus	Preclinical
PTK inhibitors	Adenovirus	Preclinical
Ribavirin *	Measles virus	Off-label clinical use
Fusion inhibitor peptides (FIPs)	Measles virus	Preclinical
Nitazoxanide *	Rubella virus	Preclinical repurposing candidate
Amphotericin B *	Rubella virus	Preclinical repurposing candidate
Pleconaril	Rhinoviruses	Completed clinical trials; not approved
Rupintrivir	Rhinoviruses	Clinical development discontinued
Soluble ICAM-1 derivatives	Rhinoviruses	Clinical/investigational

* Repurposed compounds originally approved or developed for other indications.

## Data Availability

No new data were created or analyzed in this study. Data sharing is not applicable to this article.
